# Deep learning in pulmonary nodule detection and segmentation: a systematic review

**DOI:** 10.1007/s00330-024-10907-0

**Published:** 2024-07-10

**Authors:** Chuan Gao, Linyu Wu, Wei Wu, Yichao Huang, Xinyue Wang, Zhichao Sun, Maosheng Xu, Chen Gao

**Affiliations:** 1 The First Affiliated Hospital of Zhejiang Chinese Medical University (Zhejiang Provincial Hospital of Chinese Medicine), Hangzhou, China; 2 The First School of Clinical Medicine, Zhejiang Chinese Medical University, Hangzhou, China

**Keywords:** Lung Neoplasms, Solitary pulmonary nodule, Deep learning, Tomography (X-ray computed), X-ray computed

## Abstract

**Objectives:**

The accurate detection and precise segmentation of lung nodules on computed tomography are key prerequisites for early diagnosis and appropriate treatment of lung cancer. This study was designed to compare detection and segmentation methods for pulmonary nodules using deep-learning techniques to fill methodological gaps and biases in the existing literature.

**Methods:**

This study utilized a systematic review with the Preferred Reporting Items for Systematic Reviews and Meta-Analyses guidelines, searching PubMed, Embase, Web of Science Core Collection, and the Cochrane Library databases up to May 10, 2023. The Quality Assessment of Diagnostic Accuracy Studies 2 criteria was used to assess the risk of bias and was adjusted with the Checklist for Artificial Intelligence in Medical Imaging. The study analyzed and extracted model performance, data sources, and task-focus information.

**Results:**

After screening, we included nine studies meeting our inclusion criteria. These studies were published between 2019 and 2023 and predominantly used public datasets, with the Lung Image Database Consortium Image Collection and Image Database Resource Initiative and Lung Nodule Analysis 2016 being the most common. The studies focused on detection, segmentation, and other tasks, primarily utilizing Convolutional Neural Networks for model development. Performance evaluation covered multiple metrics, including sensitivity and the Dice coefficient.

**Conclusions:**

This study highlights the potential power of deep learning in lung nodule detection and segmentation. It underscores the importance of standardized data processing, code and data sharing, the value of external test datasets, and the need to balance model complexity and efficiency in future research.

**Clinical relevance statement:**

Deep learning demonstrates significant promise in autonomously detecting and segmenting pulmonary nodules. Future research should address methodological shortcomings and variability to enhance its clinical utility.

**Key Points:**

- *Deep learning shows potential in the detection and segmentation of pulmonary nodules.*
- *There are methodological gaps and biases present in the existing literature.*
- *Factors such as external validation and transparency affect the clinical application*.

## Introduction

Lung cancer is responsible for 18.4% of all cancer deaths globally and is the leading cause of cancer-related mortality worldwide [1]. Pulmonary nodules, often an early indicator of lung cancer, do not always indicate malignancy [2]. Early detection and precise segmentation of lung nodules are crucial for accurately diagnosing and treating lung cancer [3–6].

Chest computed tomography (CT) is widely employed for pulmonary nodule detection [7, 8]. However, a single CT scan can yield hundreds of images, demanding substantial time and effort for radiologists to analyze [9]. Prolonged and arduous radiological analysis can compromise diagnostic accuracy [10, 11]. Image segmentation enables the analysis of phenotypic characteristics such as shape, size, and texture of regions of interest [12]. This examination can help determine the malignant potential. Such information is valuable for developing diagnostic and predictive models for personalized medicine in lung cancer research, leading to advancements in precision medicine within this field [13–16]. However, Image segmentation is predominantly a manual or semi-automated process, requiring labor while being prone to inter- and intra-rater discrepancies [17]. Consequently, the manual detection and segmentation of suspicious lesion regions in CT scans represent a laborious task for radiologists. In contrast, automatic detection and segmentation provide results directly from the input image, offering simplicity, speed, efficiency, and removing operator bias during segmentation [18].

Deep learning (DL), a subset of artificial intelligence (AI), leverages high-sensitivity detection, multifaceted information mining, and high-throughput computing, rendering it immensely promising in medicine [19–23]. DL has found applications in lung cancer imaging, encompassing tumor detection [24, 25], CT image segmentation [26], and classification [27, 28]. Nevertheless, most studies have focused on individual tasks, such as detection, segmentation, classification, or prognosis, and have exhibited varying degrees of quality. Additionally, no known analysis has been conducted on the quality of deep-learning studies for simultaneous pulmonary nodule detection and segmentation.

Therefore, this paper aims to conduct a comprehensive analysis and comparison of various published methods for lung nodule detection and segmentation. It seeks to summarize relevant publications to identify methodological gaps and biases. This paper will also serve as a reference for other researchers, enabling them to identify research gaps that require further investigation.

## Materials and methods

The systematic review followed the guidelines of the Preferred Reporting Items for Systematic Reviews and Meta-Analysis (PRISMA) [29]. The review was registered on PROSPERO [30] before initiation (registration No. CRD < 42023454274 >).

### Search strategy

This group searched PubMed, Embase, Web of Science Core Collection, and Cochrane Library databases up to May 10, 2023, to identify studies utilizing deep-learning techniques for detecting and segmenting lung nodules and lung cancer. Additionally, we manually searched the reference lists of relevant literature and included articles. Our search terms included “lung neoplasms,” “lung cancer,” “lung nodule,” solitary pulmonary nodule,” “multiple pulmonary nodules,” “artificial intelligence,” “deep learning,” and “image segmentation.” The detailed search criteria were described in the supplementary materials. The retrieval was performed without language and date restrictions.

### Study selection

Our study encompassed original research articles focusing on detecting and segmenting lung cancer or lung nodules using deep-learning techniques. Eligibility criteria included: (1) patients with lung cancer or lung nodules, (2) development or validation of deep-learning models for the detection and segmentation of lung nodules or cancer in CT images, and (3) the application of DL in the context of lung nodule or cancer detection and segmentation. We excluded case studies, editorials, letters, review articles, and conference abstracts. Additionally, non-deep-learning models were excluded, as well as studies exclusively employing animals or computer simulations.

### Data extraction

Two authors (Chuan G. and L.W.) independently extracted data and discussed any discrepancies. The data extraction process focused on the study parameters: the first author’s name, the year of publication, the source of the dataset, the ground truth, the focused task, and the time used for detection and segmentation. The DL parameters were also considered, including the DL algorithm, data augmentation techniques, performance measures, code/data availability, and external validation or cross-validation.

### Risk of bias assessment

Data from each included article underwent independent assessment by two radiologists (Chuan G. and L.W.). We employed the Quality Assessment of Diagnostic Accuracy Studies tool-2 (QUADAS-2) framework [31] to assess the risk of bias and applicability for each selected study, which was revised to incorporate elements from the Checklist for Artificial Intelligence in Medical Imaging (CLAIM) [32]. The detailed evaluation criteria were provided in supplementary materials. Conflict resolution occurred through consensus with a third reviewer (Chen G).

## Results

### Study selection

**Fig. 1:**
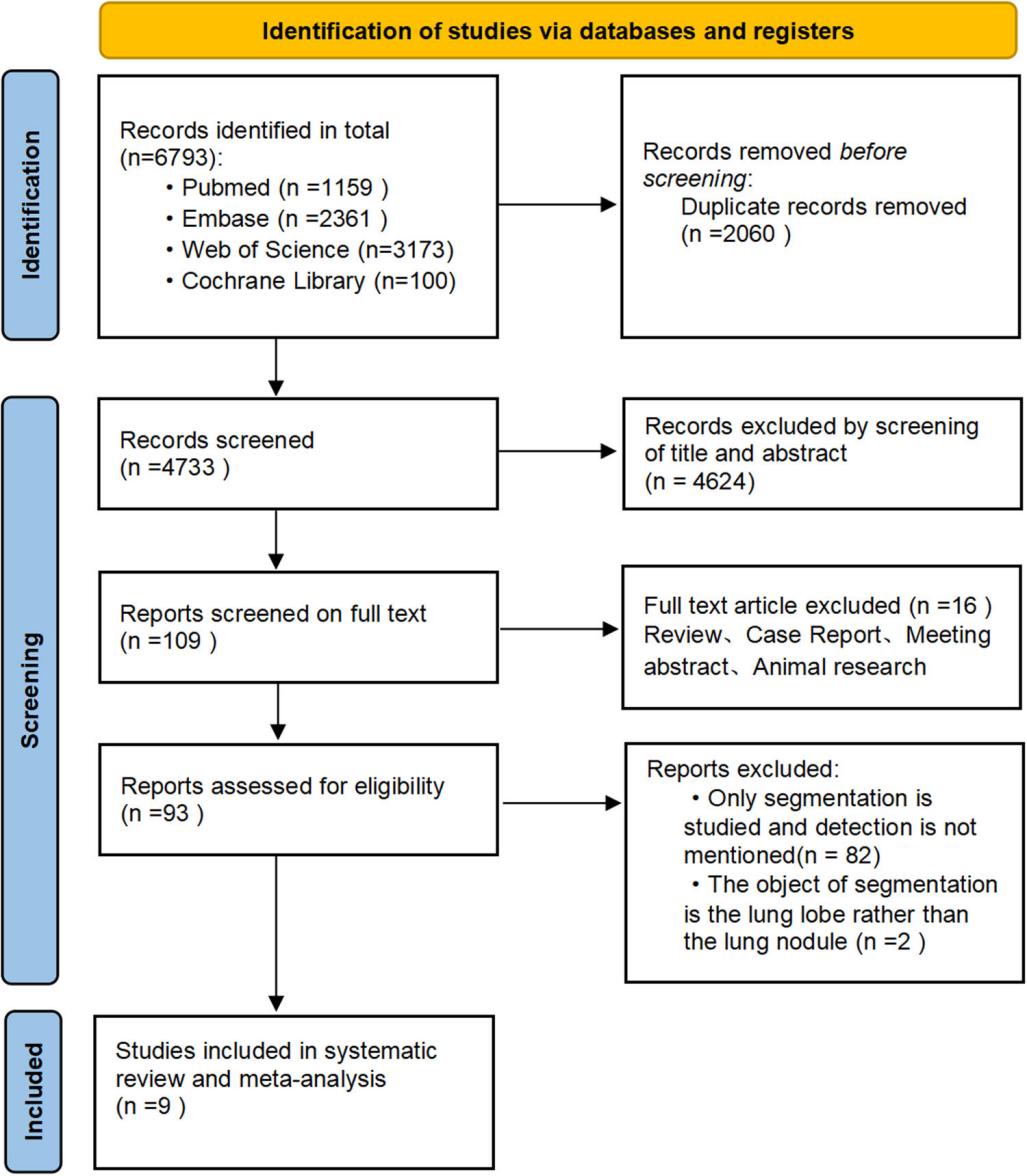
Flowchart of the study screening and selection process of this systematic review

A total of 6793 articles were retrieved for this systematic review, and two researchers conducted a full-text review of 109 articles, excluding 16 records classified as reviews, case reports, meeting abstracts, or animal research. Additionally, 82 articles were excluded for solely focusing on segmentation without detection. Two other articles were excluded as they targeted the pulmonary lobes instead of nodules. Finally, nine studies were included in this systematic review [33–41] (Fig. 1). Detailed characteristics of the included articles are presented in Tables 1 and  2. A summary of the methodological overview of the included articles is presented in Supplementary Table 1.

**Table 1:** The basic characteristics of the included studies

First author	Year of publication	Dataset	Focused task	Develop or apply	Annotation tool	Time used for detection/segmentation
Huang X [33]	2019	LIDC-IDRI LUNA16	Detection and segmentation	Develop	Ambiguity	15.319 ± 8.126 s/0.917 ± 0.835 s
Cai L [34]	2020	LUNA16 Ali TianChi challenge	Detection, segmentation, and 3D visualization	Develop	Labelme	172.9 s
Banu SF [37]	2021	LIDC-IDRI LUNA16	Detection and segmentation	Develop	Ambiguity	10.8 ms
Dutande P [35]	2021	LIDC-IDRI LNDb ILCID	Segmentation, detection, and classification	Develop	Ambiguity	Not mentioned
Zhang X [36]	2021	LIDC	Detection and segmentation	Develop	Ambiguity	Not mentioned
Hesamian MH [38]	2020	LIDC	Detection and segmentation	Develop	Ambiguity	Not mentioned
Primakov SP [39]	2022	Ten databases from different hospitals	Detection and volumetric segmentation, Prognostic	Develop and apply, prospective clinical trial	MIM version 7.0.4	2.78 ± 0.44 s
Zhou Z [40]	2022	LUNA16 LIDC-IDRI	Detection and segmentation, Classification	Develop	Ambiguity	20–60 S (960 M/GPU) 1–5 S (Titan RTX/GPU)
Dlamini S [41]	2023	TCIA LIDC-IDRI	Detection, segmentation, and 3D reconstruction	Develop	Ambiguity	11 s

*LIDC-IDRI* the lung image database consortium image collection and image database resource initiative, *LUNA16* lung nodule analysis 2016, *LNDb* the lung nodule database, *ILCID* the indian lung CT image database, *TCIA* the cancer imaging archive

**Table 2:** Deep-learning parameters

First author	External validation & cross-validation	Deep learning-related algorithm (main)	Ground truth definer	Main performance metric (detection)	Main performance metric (segmentation)	Data augmentation	Data availability	Code availability
Huang X [33]	Not mentioned	CNNs; Faster R-CNN; Modified FCN	Ambiguity	FROC curve; CPM	DSC; IOU	No	Publicly available datasets	None
Cai L [34]	Independent TianChi dataset	Mask R-CNN; FPN; RPN	Original image labeled with labelme tool	Recall (SEN); Precision; *F*-score	AP@50 AP@75	Yes	Publicly available datasets	None
Banu SF [37]	/	Faster R-CNN; AWEU-Net	Ambiguity	AP	ACC; SEN; SPE; DSC; IOU	Yes	Publicly available datasets	None
Dutande P [35]	/	SquExUNet; 3D-NodNet	Annotations from an XML file to generate segmentation ground truth masks	SEN	DSC	Yes	Publicly available datasets	None
Zhang X [36]	5-fold cross-validation	DenseNet	The intersection area of at least three radiologists’ annotations	AUC; SEN; SPE; ACC; FPR	JI; DSC; HSD; Under-segmentation rate; Over-segmentation rate	Yes	Publicly available datasets	None
Hesamian MH [38]	10-fold cross-validation	Modified U-Net	Ambiguity	/	DCS; SEN; PPV	Yes	Publicly available datasets	None
Primakov SP [39]	Datasets 8-10 (236 patients)	2D U-Net convolutional neural network (CNN)	Contours segmented by experts	ROC; AUC; confusion matrix; Sensitivity; specificity	DSC; JI; APL; H95th; Surf DSC	Yes	Part of it is available (datasets 1; 6; 7; 8)	Yes
Zhou Z [40]	/	2D-based Yolov5 CNS algorithm; 3D-based U-Net	Standard physician annotations	SEN; CPM	DSC; recall; precision	Yes	Publicly available datasets	None
Dlamini S [41]	/	YOLOv4	Manually prepared by professional clinicians and verified by a thoracic surgeon	IOU; Precision; SEN; F1 score; mAP@.5	DSC	Yes	Data will be made available on request	None

*ACC* accuracy, *AP* average precision, *AP@50* average precision at 50, *AP@75* average precision at 75, *APL* added path length, *AUC* area under receiver operating characteristic curve, *AWEU-Net* attention-aware weight excitation U-Net, *CNS* candidate nodule selection, *CNNS* convolutional neural networks, *CPM* competition performance metric, *DenseNet* densely connected convolutional network, *DSC* dice similar coefficient, *Faster R-CNN* faster region-based convolutional neural networks, *FCN* fully convolutional network, *FPN* feature pyramid network, *FPR* false positive rate, *F-score(F1 score)* *F*-measure score, *FROC curve* free-response receiver operating characteristic curve, *HSD(HD)* Hausdorff distance, *H95th* 95th percentile Hausdorff distance, *IOU* intersection over Union, *JI* Jaccard index, *Mask R-CNN* mask region-based convolutional neural network, *mAP* mean average precision, *mAP@.5* mean average precision at 5, *PPV* positive predictive value, *ROC* receiver operating characteristic curve, *RPN* region proposal network, *SEN* sensitivity, *SPE* specificity, *SquExUNet* squeeze and excitation U-Net, *Surf DSC* surface DSC

### Study characteristics

Table 1 summarizes the characteristics of the nine studies included in this analysis. These studies were published between 2019 and 2023, with one (1/9, 11%) [39] being a prospective in-silico clinical trial, while the rest (8/9, 89%) were retrospective in design. Among the nine articles, eight (8/9, 89%) [33–38, 40, 41] made use of publicly available datasets, with the Lung Image Database Consortium Image Collection and Image Database Resource Initiative (LIDC-IDRI) [42] dataset and its subsidiary, Lung Nodule Analysis 16 (LUNA16) [43], emerging as the most frequently employed datasets. Primary tasks addressed by most studies [33–41] encompassed detection and segmentation, while two (2/9, 22%) studies [34, 41] included three-dimensional (3D) visualization reconstruction. Two (2/9, 22%) [35, 40] studies incorporated classification, while another article (1/9, 11%) [39] incorporated prognosis prediction. Out of the nine studies, eight (8/9, 89%) [33–38, 40, 41] focused on the development of DL models or architectures, while one study (1/9, 11%) [39] focused on developing and building an application or a working prototype. Furthermore, six (2/3, 67%) [33, 34, 37, 39–41] of the nine studies reported the inference time for detection and segmentation tasks.

### Ground truth definition

The definition of ground truth was ambiguous in three articles (1/3, 33%) [33, 37, 38]. One (1/9, 11%) [34] article defined raw images labeled with the label tool as ground truth, while another (1/9, 11%) [35] research mentioned the utilization of annotations in XML files to generate segmentation ground truth masks. One (1/9, 11%) article [36] considered intersection regions annotated by at least three radiologists as the ground truth. Similarly, another paper (1/9, 11%) [39] treated the contours segmented by experts as ground truth. One (1/9, 11%) article [40] also referred to the ground truth determined by standard physician annotations. Lastly, one article (1/9, 11%) [41] defined the ground truth as being manually prepared by expert clinicians and reviewed by thoracic surgeons.

### External validation or cross-validation and data augmentation

Among the nine studies, two (2/9, 22%) [34, 39] conducted external validation, while one (1/9, 11%) [36] employed 5-fold cross-validation, and another (1/9, 11%) [38] utilized 10-fold cross-validation. Out of the nine articles, eight (8/9, 89%) [34–41] employed data augmentation technology, with only one (1/9, 11%) [33] article not utilizing it.

### Deep-learning algorithms

A diverse range of deep-learning algorithms incorporated by the articles included in the systematic review can be categorized into distinct types, including models tailored for object classification and detection, such as densely connected convolutional networks (DenseNet) [36], YOLOv4 and YOLOv5 [40, 41], as well as models designed for image segmentation tasks, like U-Net [37–40], Fast region-based convolutional neural network (R-CNN) [33, 37], and Mask R-CNN [34]. Furthermore, certain studies have employed general-purpose architectures, such as region-based convolutional neural networks (FCN) and feature pyramid networks (FPN) [33, 34], for various image analysis applications.

### Code and Data Availability

Among the nine studies, 7 (7/9, 78%) [33–38, 40] used open-source public datasets. In one (1/9, 11%) study [39], a portion of the data was accessible; in another (1/9, 11%) study [41], it was mentioned that the data would be provided upon request. Out of the nine publications, only one (1/9, 11%) [39] author has uploaded the code to a public repository, while the remainder (8/9, 89%) does not mention the availability of code.

### Performance metrics

Table 3 presents a summary of the performance metrics observed in the studies. The performance indicators for nodule detection include accuracy (ACC), sensitivity (SEN), precision, *F*-score, and area under the receiver operating characteristic curve (AUC). As for nodule segmentation, the reported performance metrics encompassed dice similar coefficient (DSC), Jaccard index (JI), Hausdorff distance (HD), and Intersection over Union (IOU). The accuracy of pulmonary nodule detection in these studies exceeded 90% [33, 36, 37], and the detection sensitivity was as high as 97% [39, 41]. In addition, the DSC of pulmonary nodule segmentation can be as high as 0.93 [38].

**Table 3:** Main performance metrics

First author	Detection	Segmentation
Huang X [33]	Accuracy: 91.4% (1FPs)/94.6% (4FPs) CPM: 0.866/0.875/0.880	DSC: 0.793 ± 0.082 IOU: 70.24 ± 12.04%
Cai L [34]	Sensitivity: 88.1% (1FPs)/88.7% (4FPs) Precision: 0.486 score: 0.628 CPM: 0.796/0.625	AP@50: 0.882 AP@75: 0.571
Banu SF [37]	Accuracy: 0.9132/0.9466 Specificity: 0.9346/0.9641 Sensitivity: 90.84%/91.69% AP: 91.44%/92.67%	DSC: 89.79%/90.35% IOU: 82.34%/83.21%
Dutande P [35]	Sensitivity: 90.01%	DSC: 0.80
Zhang X [36]	AUC: 0.98 Sensitivity: 0.7936 ± 0.1417 Specificity: 0.9998 ± 0.0003 Accuracy: 0.9997 ± 0.0003 False positive rate: 0.0002 ± 0.0003	JI: 0.6385 ± 0.1309 DSC: 0.7710 ± 0.1057, HSD: 3.5123 ± 3.1251 Under-segmentation rate: 0.1769 ± 0.1308 Over-segmentation rate: 0.1848 ± 0.1463
Hesamian MH [38]	/	DSC: 93.14 ± 0.27% SEN: 91.76 ± 1.1% PPV: 93.3 ± 0.8%
Primakov SP [39]	AUC: 0.98 Specificity: 0.99 Sensitivity: 0.97	DSC: 0.82 JI: 0.70 H95th: 9.43 mm Surf DSC: 0.63 APL: 306 cm
Zhou Z [40]	Sensitivity: 95.95% CPM:89.5%	DSC: 86.75 ± 9.25%
Dlamini S [41]	Precision: 96.57% Sensitivity: 97.02% F1 score: 96.79% mAP@.5: 88.76% IOU: 92.34%	DSC: 92.19%

*FPs* false positives, *AUC* area under receiver operating characteristic curve, *DSC* dice similar coefficient, *HSD* Hausdorff distance, *JI* Jaccard index, *IOU* intersection over Union, *F-score(F1 score)* *F*-measure score, *AP* average precision, *AP@50* average precision at 50, *AP@75* average precision at 75, *mAP* mean average precision, *mAP@.5* mean average precision at 5, *PPV* positive predictive value, *CPM* competition performance metric, *Surf DSC* surface DSC, *H95th* 95th percentile Hausdorff distance, *APL* added path length

### Risk of bias and quality assessment

**Fig. 2:**
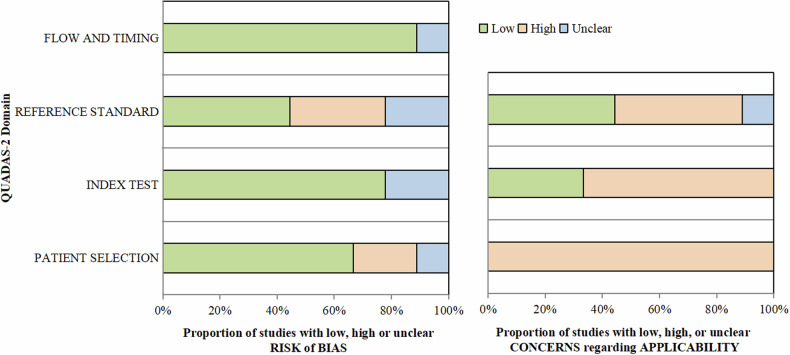
Graphical representation of the Quality Assessment of Diagnostic Accuracy Studies version 2 (QUADAS-2) assessment results of included studies. Stacked bar charts show the results of the quality assessment for risk of bias and applicability of included studies. QUADAS-2 scores for methodologic study quality are expressed as the percentage of studies that met each criterion. For each quality domain, the proportion of included studies that were determined to have low, high, or unclear risk of bias and/or concerns regarding applicability is displayed in green, orange, and blue, respectively. QUADAS-2, Quality Assessment of Diagnostic Accuracy Studies 2

Table 4 and Fig. 2 present the quality assessment of the included studies. Among the seven items of the QUADAS-2 tool, the most common item that could have been improved was the applicability concern regarding patient selection. In terms of the risk of bias, 7 out of 9 (7/9, 78%) studies were determined to have a low risk of bias in the domain of “*index test*” [33–37, 39, 40], and 8 out of 9 (8/9, 89%) studies were judged to have a low risk of bias in terms of “*flow and timing*” [33–38, 40, 41]. Additionally, 6 out of 9 (2/3, 67%) studies were found to have a low risk of bias in terms of “*patient selection*” [33–37, 40]. However, only 44% of the studies (4/9) [36, 39–41] were considered to have a low risk of bias in the “*reference standard*” domain. Only two (2/9, 22%) articles [36, 40] were determined to have a low risk of bias in all four domains.

**Table 4:**
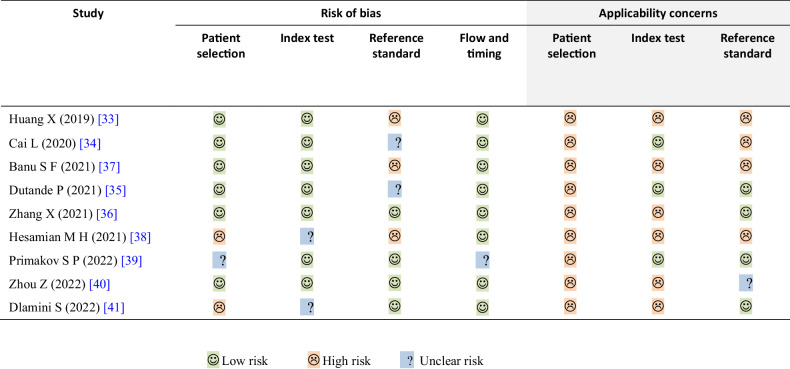
Quality Assessment of Diagnostic Accuracy Studies version 2 (QUADAS-2) assessment results of included studies

## Discussion

**Fig. 3:**
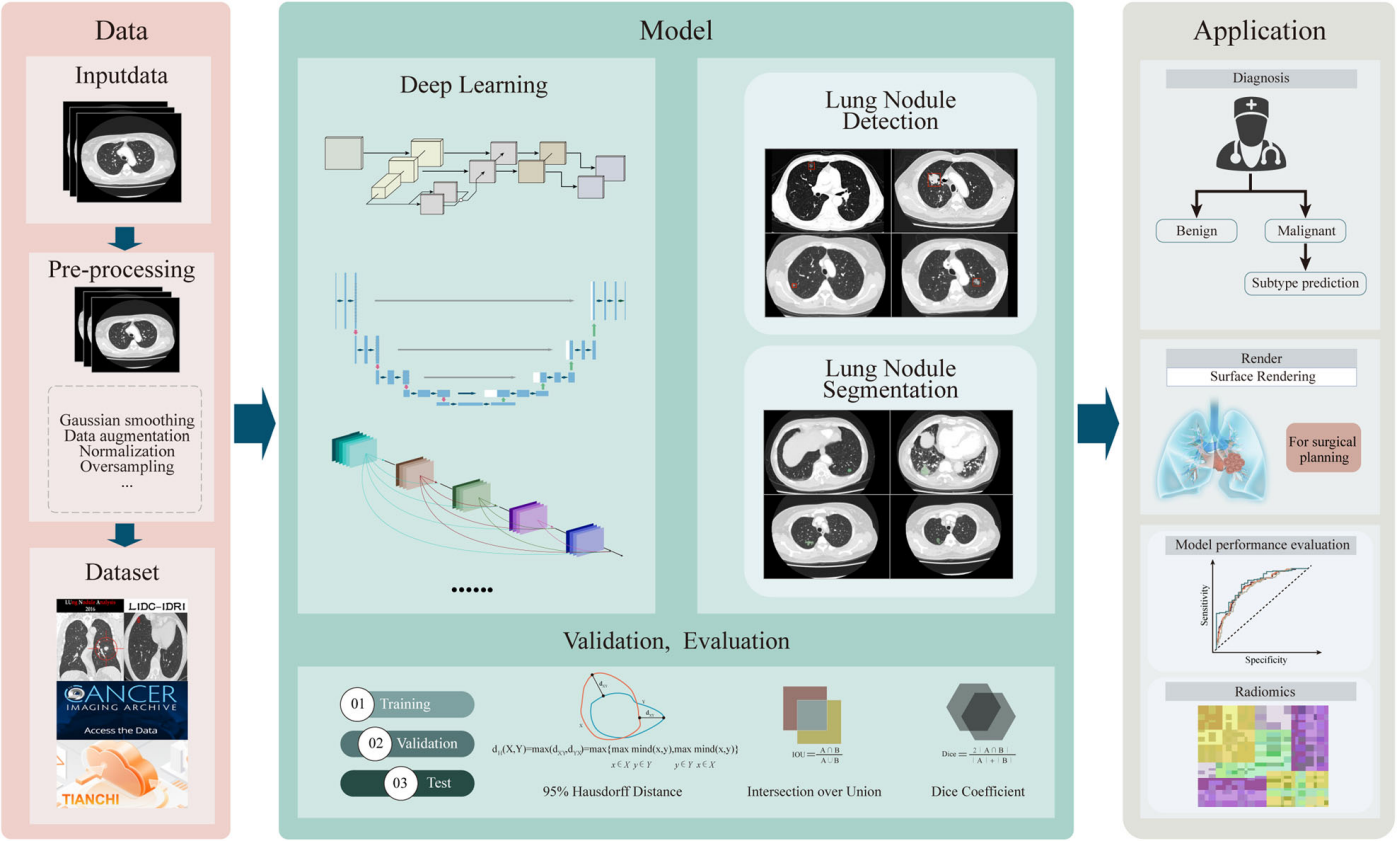
Deep learning in lung nodule detection and segmentation

This systematic review analyzed nine pertinent articles from four prominent databases and found that DL shows significant potential in detecting and segmenting pulmonary nodules concurrently instead of being restricted to a singular task [44–49] (Fig. 3). Nevertheless, the analysis identified methodological shortcomings and variations among the studies included.

Accurate detection is vital for precise segmentation. Some studies have successfully implemented multi-task learning [50–52], demonstrating the possibility of solving both aspects simultaneously. Human experts’ manual detection and annotation of lung cancer nodules is a time-consuming and inconsistent process [17, 53]. In contrast, deep-learning algorithms have demonstrated the ability to perform the same task rapidly and consistently [33, 34, 37, 39–41].

The quality of the generated automatic segmentation was evaluated against the corresponding reference segmentation, known as *ground truths*, demonstrating adherence to the requirements and best practices outlined in the AI Checklist [32]. The requirements included providing a detailed definition of the reference standard and a rationale for its selection [54, 55]. It was also important to mention the source of the ground truth annotation and the qualifications of the annotator. Additionally, it was necessary to measure annotation tools and internal variability and propose to mitigate and resolve any differences. However, the methodologies employed in the reviewed studies varied and were not standardized. Although four studies [36, 39–41] mentioned the involvement of physicians or experts in providing basic facts, most studies did not specify the professional background of these experts, such as whether they were experienced radiologists, trainees, or other non-clinical researchers. Most studies did not clearly state the number of relevant experts involved [33–35, 37–40] or the annotation tool used [33, 35–38, 40, 41]. Differences in the focus of the publishing journal and the authors’ background, such as computing researchers versus clinicians, may contribute to this variation. Furthermore, certain studies utilized public datasets where the ground truth is established by other research teams or experts, limiting the authors’ ability to provide detailed explanations.

Eight out of nine studies utilized public datasets, the most frequently used ones being the LIDC-IDRI [42] and the LUNA16 dataset [43]. Public datasets offer abundant annotated data for training deep-learning models and enable researchers to validate and compare algorithms, enhancing research reproducibility [56, 57]. However, both LIDC/IDRI and LUNA16 originated from the United States and may not fully represent the global population. Additionally, these datasets often have an unbalanced male:female ratio, potentially hindering comprehensive gender-based analyses. While these datasets contain both standard-dose diagnostic and low-dose CT scans for lung cancer screening, the latter may compromise image quality and nodule contrast, presenting challenges in nodule detection and segmentation. The limitations of public datasets need to be carefully considered during model development and research to ensure the quality and representativeness of the data.

Furthermore, all studies have embraced convolutional neural networks (CNNs) and their derivatives, progressively enhancing network depth and complexity to bolster feature extraction capabilities [33–41]. Escalating model depth necessitates augmented computational resources, leading to longer training durations [58, 59]. To mitigate prolonged training, some algorithms have resorted to strategies that entail sacrificing substantial graphics processing unit (GPU) memory [60, 61]. However, this approach is unsustainable in the long run. Therefore, balancing network design, computation time, and cost is imperative.

Despite the growing interest in open science in current scientific research, only one study [39] has made its code openly accessible. Many research teams are reluctant to share their project code because of concerns about intellectual property and commercial interests. However, sharing project code can enhance the reproducibility of research findings and facilitate the validation of existing results, ultimately fostering collaboration within the scientific research community, and advancing the frontiers of knowledge.

Only two studies [34, 39] in our research utilized external test datasets, highlighting the inherent challenge of obtaining data from external collections. Some strategies have been employed to address this issue. For instance, two studies [36, 38] employed cross-validation methods, partially compensating for the lack of external testing datasets [62]. However, the absence of external validation datasets remains a significant limitation for the clinical applicability of the developed models [63]. This limitation arises from the inability of most studies to assess the risk of overfitting. Cross-validation helps mitigate this by evaluating model performance and detecting overfitting within existing data through repeated training and validation on subsets. However, its computational cost is high and there are problems such as sample imbalance. In addition, its results may be affected by the randomness of data partitioning. Different data partitioning may lead to different model performance evaluation results, thus affecting the accurate evaluation of the model. A combination of cross-validation and external validation was essential to achieve a more comprehensive and reliable evaluation of deep-learning models. Only one [33] article did not employ data augmentation techniques. Data augmentation is a highly effective method for enhancing data heterogeneity, preventing overfitting, and improving the robustness of CNN networks [64, 65].

The included studies have mostly concentrated on developing DL models and architectures [33–38, 40, 41]. However, only one study has examined the clinical application of these techniques [39]. This indicated the limited experience of non-medical researchers in selecting clinically relevant outcomes and their limited applicability in clinical practice.

Our study included only nine articles, while a wide variety of chest computer-aided diagnosis (CAD) systems are currently available on the market [66]. This disparity in numbers can be traced back to the progression of medical imaging CAD systems, transitioning from basic image-processing techniques to more advanced machine learning and deep-learning algorithms [67]. While some systems in the market still rely on traditional image-processing and machine-learning methods, the incorporation of DL has significantly enhanced the accuracy and efficiency of CAD systems [68]. Commercializing CAD systems involves various manufacturers and development teams, each capable of creating unique chest CAD systems based on their research and technological advancements [69]. These systems may consist of individual detection or segmentation modules customized to meet specific user requirements, leading to a diverse range of systems despite the limited number of articles.

This systematic review has several notable limitations. Firstly, the number of eligible studies was relatively small. Secondly, a meta-analysis of pooled outcomes could not be conducted as the median and standard deviation of the five articles were not provided in the results. Thirdly, the evaluation did not include individuals from engineering backgrounds, which may introduce a certain level of professional bias. Additionally, by excluding articles published in conference proceedings, there is a chance of excluding promising methods for lung nodule detection and segmentation.

## Conclusions

In conclusion, this systematic review emphasizes the increasing significance of DL in detecting and segmenting lung nodules, which are crucial for early lung cancer diagnosis. Ensembles of deep-learning models demonstrate considerable potential in expediting the detection and segmentation processes, offering substantial advantages in clinical practice. However, certain challenges persist, including the need for diverse models, external validation, efficiency, and transparency. Future endeavors should address these challenges to foster the advancement of DL in lung cancer imaging, ultimately enhancing the accuracy and efficiency of early diagnosis.

## Supplementary information

ELECTRONIC SUPPLEMENTARY MATERIAL

## Data Availability

All data generated or analysed during this study are included in this published article [and its supplementary information files].

## Abbreviations

ACC: Accuracy
AI: Artificial intelligence
AUC: Area under the receiver operating characteristic curve
CAD: Computer-aided diagnosis
CNN: Convolutional neural network
CNNS: Convolutional neural networks
CT: Computed tomography
DenseNet: Densely connected convolutional network
DL: Deep learning
DSC: Dice similar coefficient
GPU: Graphics processing unit
IOU: Intersection over Union
JI: Jaccard index
LIDC-IDRI: The Lung Image Database Consortium Image Collection and Image Database Resource Initiative
LUNA16: Lung Nodule Analysis 2016
QUADAS-2: Quality Assessment of Diagnostic Accuracy Studies 2
R-CNN: Region-based convolutional neural network
SEN: Sensitivity
