# Supplementary material for: Deep learning in pulmonary nodule detection and segmentation: a systematic review
Source: Eur Radiol. 2024 Jul 10;35(1):255–66. doi: 10.1007/s00330-024-10907-0 (PMC11632000; doi:10.1007/s00330-024-10907-0)
Supplement: Supplementary file 1 — ELECTRONIC SUPPLEMENTARY MATERIAL [file 330_2024_10907_MOESM1_ESM.docx]

**Supplementary Material**

**Article title:**

Deep Learning in Pulmonary Nodule Detection and Segmentation: A Systematic Review

**Journal name:**

European Radiology

**Search Strategy:**

**1.PubMed search strategy (n = 1159 )**

#1

(((((((((((Lung Neoplasms[MeSH Terms]) OR (bronchial neoplasms[MeSH Terms])) OR (Carcinoma, Bronchogenic[MeSH Terms])) OR (Small Cell Lung Carcinoma[MeSH Terms])) OR (Carcinoma, Small Cell[MeSH Terms])) OR (Solitary Pulmonary Nodule[MeSH Terms])) OR (Multiple Pulmonary Nodules[MeSH Terms])) OR (Respiratory Tract Neoplasms[MeSH Terms])) OR (Adenocarcinoma of Lung[MeSH Terms])) OR (Adenocarcinoma, Bronchiolo-Alveolar[MeSH Terms])) OR (Tracheal Neoplasms[MeSH Terms])) OR (Carcinoma, Non-Small-Cell Lung[MeSH Terms])

#2 ((((((((((((((((((((((((((((((((((((((((((((((((((((((((((((((((((((((((((((((((((((((((((((((((((((((((((((((((((((((((((((((((((((((((((((((((((((((((((((((((((((((((((((((((((((((((((((((((((((((((((((((((((((((((((((((((((((((((((((((((((((((((((((((((((((((((((((((((Adenocarcinoma, Lung[Title/Abstract]) OR (Adenocarcinomas, Lung[Title/Abstract])) OR (Adenocarcinoma, Bronchiolo-Alveolar[Title/Abstract])) OR (Adenocarcinoma, Bronchiolo Alveolar[Title/Abstract])) OR (Adenocarcinomas, Bronchiolo-Alveolar[Title/Abstract])) OR (Adenocarcinoma, Alveolar[Title/Abstract])) OR (Adenocarcinomas, Alveolar[Title/Abstract])) OR (Adenocarcinoma of Lung[Title/Abstract])) OR (Alveolar Adenocarcinoma[Title/Abstract])) OR (Alveolar Adenocarcinomas[Title/Abstract])) OR (Alveolar Carcinoma[Title/Abstract])) OR (Alveolar Carcinomas[Title/Abstract])) OR (Alveolar Cell Carcinoma[Title/Abstract])) OR (Alveolar Cell Carcinomas[Title/Abstract])) OR (alveobronchial carcinoma[Title/Abstract])) OR (alveolobronchiolar carcinoma[Title/Abstract])) OR (alveolar cell cancer[Title/Abstract])) OR (alveolar clear cell sarcoma[Title/Abstract])) OR (alveolar lung carcinoma[Title/Abstract])) OR (alveolus cell cancer[Title/Abstract])) OR (alveolus cell cancer, lung[Title/Abstract])) OR (Bronchiolo-Alveolar Carcinoma[Title/Abstract])) OR (Bronchiolo-Alveolar Carcinomas[Title/Abstract])) OR (Bronchiolo-Alveolar Adenocarcinoma[Title/Abstract])) OR (Bronchiolo-Alveolar Adenocarcinomas[Title/Abstract])) OR (Bronchial Carcinoma[Title/Abstract])) OR (Bronchial Carcinomas[Title/Abstract])) OR (Bronchogenic Carcinoma[Title/Abstract])) OR (Bronchogenic Carcinomas[Title/Abstract])) OR (Bronchiolar Carcinoma[Title/Abstract])) OR (Bronchiolar Carcinomas[Title/Abstract])) OR (Bronchioloalveolar Carcinoma[Title/Abstract])) OR (Bronchioloalveolar Carcinomas[Title/Abstract])) OR (Bronchial Neoplasm[Title/Abstract])) OR (Bronchial Neoplasms[Title/Abstract])) OR (bronchioloalveolar cell carcinoma[Title/Abstract])) OR (bronchioloalveolar carcinoma[Title/Abstract])) OR (broncho alveolar carcinoma[Title/Abstract])) OR (bronchioalveolar lung carcinoma[Title/Abstract])) OR (bronchiolar cancer[Title/Abstract])) OR (bronchiolar cell cancer[Title/Abstract])) OR (bronchiolar cell carcinoma[Title/Abstract])) OR (bronchoalveolar carcinoma[Title/Abstract])) OR (bronchoalveolar cancer[Title/Abstract])) OR (bronchial carcinomata[Title/Abstract])) OR (broncho-pulmonary carcinoma[Title/Abstract])) OR (bronchogenic carcinoma of the lung[Title/Abstract])) OR (bronchogenic lung carcinoma[Title/Abstract])) OR (bronchopulmonary carcinoma[Title/Abstract])) OR (bronchus carcinomacarcinoma, bronchiolar[Title/Abstract])) OR (bronchopulmonary cancer[Title/Abstract])) OR (broncho-pulmonary neoplasm[Title/Abstract])) OR (broncho-pulmonary tumor[Title/Abstract])) OR (bronchopulmonary neoplasia[Title/Abstract])) OR (bronchopulmonary neoplasm[Title/Abstract])) OR (bronchopulmonary tumorCa lung[Title/Abstract])) OR (bronchial small cell cancer[Title/Abstract])) OR (bronchial small cell carcinoma[Title/Abstract])) OR (bronchial non small cell cancer[Title/Abstract])) OR (bronchial non small cell carcinoma[Title/Abstract])) OR (Cancer, Lung[Title/Abstract])) OR (Cancers, Lung[Title/Abstract])) OR (Cancer, Pulmonary[Title/Abstract])) OR (Cancers, Pulmonary[Title/Abstract])) OR (Cancer of the Lung[Title/Abstract])) OR (Cancer of Lung[Title/Abstract])) OR (cancer of the respiratory tract[Title/Abstract])) OR (cancer in the thorax[Title/Abstract])) OR (cancer of the thoracic cavity[Title/Abstract])) OR (cancer of the thorax[Title/Abstract])) OR (cancer of the thorax[Title/Abstract] AND thoracic cavity[Title/Abstract])) OR (cancerous lung cell line[Title/Abstract])) OR (cancerous pulmonary cell line[Title/Abstract])) OR (Carcinoma, Alveolar[Title/Abstract])) OR (Carcinomas, Alveolar[Title/Abstract])) OR (Carcinoma, Alveolar Cell[Title/Abstract])) OR (Carcinomas, Alveolar Cell[Title/Abstract])) OR (Carcinoma, Bronchial[Title/Abstract])) OR (Carcinomas, Bronchial[Title/Abstract])) OR (Carcinoma, Bronchiolar[Title/Abstract])) OR (Carcinomas, Bronchiolar[Title/Abstract])) OR (Carcinoma, Bronchogenic[Title/Abstract])) OR (Carcinomas, Bronchogenic[Title/Abstract])) OR (Carcinoma, Bronchiolo-Alveolar[Title/Abstract])) OR (Carcinoma, Bronchiolo Alveolar[Title/Abstract])) OR (Carcinomas, Bronchiolo-Alveolar[Title/Abstract])) OR (Carcinoma, Bronchioloalveolar[Title/Abstract])) OR (Carcinomas, Bronchioloalveolar[Title/Abstract])) OR (Carcinoma, Non-Small-Cell Lung[Title/Abstract])) OR (Carcinoma, Non Small Cell Lung[Title/Abstract])) OR (Carcinomas, Non-Small-Cell Lung[Title/Abstract])) OR (Carcinoma, Non-Small Cell Lung[Title/Abstract])) OR (Carcinoma, Non-Small-Cell Lung[Title/Abstract])) OR (Carcinoma, Non Small Cell Lung[Title/Abstract])) OR (Carcinomas, Non-Small-Cell Lung[Title/Abstract])) OR (Carcinoma, Non-Small Cell Lung[Title/Abstract])) OR (Carcinoma, Oat Cell[Title/Abstract])) OR (Carcinomas, Oat Cell[Title/Abstract])) OR (Carcinoma, Small Cell Lung[Title/Abstract])) OR (Carcinoma, Small Cell[Title/Abstract])) OR (Carcinomas, Small Cell[Title/Abstract])) OR (carcinoma of the thorax[Title/Abstract])) OR (carcinoma of lung[Title/Abstract])) OR (carcinoma of the lung[Title/Abstract])) OR (carcinoma of the bronchus[Title/Abstract])) OR (carcinoma of bronchus[Title/Abstract])) OR (carcinoma, lewis[Title/Abstract])) OR (carcinoma, lewis lung[Title/Abstract])) OR (carcinoma, lung[Title/Abstract])) OR (carcinoma pulmonum[Title/Abstract])) OR (carcinomatosis of the lung[Title/Abstract])) OR (carcinogenesis in the respiratory tract[Title/Abstract])) OR (carcinogenesis of the lungbroncho-pulmonary cancer[Title/Abstract])) OR (carcinomatous lung[Title/Abstract])) OR (carcinomatous pulmonary[Title/Abstract])) OR (invasive lung carcinoma[Title/Abstract])) OR (invasive pulmonary carcinoma[Title/Abstract])) OR (Lung Adenocarcinoma[Title/Abstract])) OR (Lung Adenocarcinomas[Title/Abstract])) OR (Lung Cancer[Title/Abstract])) OR (Lung Cancers[Title/Abstract])) OR (Lung Carcinoma, Non-Small-Cell[Title/Abstract])) OR (Lung Carcinomas, Non-Small-Cell[Title/Abstract])) OR (Lung Neoplasm[Title/Abstract])) OR (Lung Neoplasms[Title/Abstract])) OR (Lung Nodule, Solitary[Title/Abstract])) OR (lung alveolar carcinoma[Title/Abstract])) OR (lung alveolus cell carcinoma[Title/Abstract])) OR (lung alveolar cell cancer[Title/Abstract])) OR (lung alveolar cell carcinoma[Title/Abstract])) OR (lung alveolus cell cancer[Title/Abstract])) OR (lung cancer cell line[Title/Abstract])) OR (lung cancer-derived cell line[Title/Abstract])) OR (lung cancerous cell line[Title/Abstract])) OR (lung cancers cell line[Title/Abstract])) OR (lung carcinoma[Title/Abstract])) OR (lung carcinomata[Title/Abstract])) OR (lung carcinomatosis[Title/Abstract])) OR (lung cavitary carcinoma[Title/Abstract])) OR (lung carcinogenesis[Title/Abstract])) OR (lung cancerogenesismicrocellular lung carcinoma[Title/Abstract])) OR (lung cancermalignancies of the lung[Title/Abstract])) OR (lung cancer, non small cell[Title/Abstract])) OR (lung malignancy[Title/Abstract])) OR (lung malignancies[Title/Abstract])) OR (lung neoplasia[Title/Abstract])) OR (lung non small cell cancer[Title/Abstract])) OR (lung non small cell carcinoma[Title/Abstract])) OR (lung oat cell carcinoma[Title/Abstract])) OR (lung small cell carcinoma[Title/Abstract])) OR (lung small cell cancer[Title/Abstract])) OR (lung tumor[Title/Abstract])) OR (lung tumour[Title/Abstract])) OR (lung tumorigenesis[Title/Abstract])) OR (lewis lung carcinoma[Title/Abstract])) OR (lewis lung tumor[Title/Abstract])) OR (lewis lung tumour[Title/Abstract])) OR (Multiple Pulmonary Nodule[Title/Abstract])) OR (Multiple Pulmonary Nodules[Title/Abstract])) OR (malignancy of the lung[Title/Abstract])) OR (malignant lung neoplasm[Title/Abstract])) OR (malignant lung tumor[Title/Abstract])) OR (malignant neoplasm of the lung[Title/Abstract])) OR (malignant tumor of the lung[Title/Abstract])) OR (malignancies of the respiratory tract[Title/Abstract])) OR (malignant lung cell line[Title/Abstract])) OR (malignant pulmonary cell line[Title/Abstract])) OR (Neoplasm, Bronchial[Title/Abstract])) OR (Neoplasms, Bronchial[Title/Abstract])) OR (Neoplasm, Lung[Title/Abstract])) OR (Neoplasms, Lung[Title/Abstract])) OR (Non-Small Cell Lung Cancer[Title/Abstract])) OR (Non Small Cell Lung Carcinoma[Title/Abstract])) OR (Non-Small-Cell Lung Carcinoma[Title/Abstract])) OR (Non-Small-Cell Lung Carcinomas[Title/Abstract])) OR (Non-Small Cell Lung Carcinoma[Title/Abstract])) OR (Non-Small-Cell Lung Carcinoma[Title/Abstract])) OR (Non-Small Cell Lung Carcinoma[Title/Abstract])) OR (Nonsmall Cell Lung Cancer[Title/Abstract])) OR (non oat cell lung cancer[Title/Abstract])) OR (non small cell bronchial cancer[Title/Abstract])) OR (non small cell cancer, lung[Title/Abstract])) OR (non small cell pulmonary cancer[Title/Abstract])) OR (non small cell pulmonary carcinoma[Title/Abstract])) OR (non squamous NSCLC[Title/Abstract])) OR (nonsmall cell carcinoma of the lung[Title/Abstract])) OR (nonsmall cell lung carcinoma[Title/Abstract])) OR (Nodule, Solitary Lung[Title/Abstract])) OR (Nodule, Solitary Pulmonary[Title/Abstract])) OR (Nodules, Solitary Pulmonary[Title/Abstract])) OR (neoplasia of the lung[Title/Abstract])) OR (neoplastic lung[Title/Abstract])) OR (Oat Cell Carcinoma of Lung[Title/Abstract])) OR (Oat Cell Lung Cancer[Title/Abstract])) OR (Oat Cell Carcinoma[Title/Abstract])) OR (Oat Cell Carcinomas[Title/Abstract])) OR (oat cell cancer of the lung[Title/Abstract])) OR (oat cell carcinoma of the lung[Title/Abstract])) OR (oat cell lung carcinoma[Title/Abstract])) OR (Pulmonary Cancer[Title/Abstract])) OR (Pulmonary Cancers[Title/Abstract])) OR (Pulmonary Neoplasm[Title/Abstract])) OR (Pulmonary Neoplasms[Title/Abstract])) OR (Pulmonary Nodule, Solitary[Title/Abstract])) OR (Pulmonary Nodules, Solitary[Title/Abstract])) OR (Pulmonary Nodule, Multiple[Title/Abstract])) OR (Pulmonary Nodules, Multiple[Title/Abstract])) OR (pulmonary alveolar cell cancer[Title/Abstract])) OR (pulmonary alveolus cell cancer[Title/Abstract])) OR (pulmonary cancer cell line[Title/Abstract])) OR (pulmonary cancerogenesis[Title/Abstract])) OR (pulmonary carcinogenesis[Title/Abstract])) OR (peribronchial carcinoma[Title/Abstract])) OR (pulmonary carcinoma[Title/Abstract])) OR (pulmonary carcinomatosis[Title/Abstract])) OR (pulmonary malignancies[Title/Abstract])) OR (pulmonary malignancy[Title/Abstract])) OR (pulmonary neoplasia[Title/Abstract])) OR (pulmonary non small cell cancer[Title/Abstract])) OR (pulmonary non small cell carcinoma[Title/Abstract])) OR (pulmonary small cell cancer[Title/Abstract])) OR (pulmonary small cell carcinomapulmonary tumor[Title/Abstract])) OR (pulmonary tumorigenesis[Title/Abstract])) OR (pulmonary tumour[Title/Abstract])) OR (Respiratory Tract Neoplasms[Title/Abstract])) OR (Respiratory Tract Neoplasm[Title/Abstract])) OR (Respiratory System Neoplasms[Title/Abstract])) OR (Respiratory System Neoplasm[Title/Abstract])) OR (Respiratory Neoplasms[Title/Abstract])) OR (Respiratory Neoplasm[Title/Abstract])) OR (respiratory tract cancer[Title/Abstract])) OR (respiratory cancer[Title/Abstract])) OR (respiratory carcinogenesis[Title/Abstract])) OR (respiratory malignancy[Title/Abstract])) OR (respiratory malignancies[Title/Abstract])) OR (respiratory tract carcinogenesis[Title/Abstract])) OR (respiratory tract malignancies[Title/Abstract])) OR (respiratory tract malignancy[Title/Abstract])) OR (respiratory tract carcinoma[Title/Abstract])) OR (respiratory system carcinoma[Title/Abstract])) OR (Small Cell Lung Cancer[Title/Abstract])) OR (Small Cell Cancer Of The Lung[Title/Abstract])) OR (Small Cell Carcinoma[Title/Abstract])) OR (Small Cell Carcinomas[Title/Abstract])) OR (Small Cell Lung Carcinoma[Title/Abstract])) OR (Solitary Pulmonary Nodule[Title/Abstract])) OR (Solitary Pulmonary Nodules[Title/Abstract])) OR (Solitary Lung Nodule[Title/Abstract])) OR (Solitary Lung Nodules[Title/Abstract])) OR (small cell bronchial cancer[Title/Abstract])) OR (small cell bronchial carcinoma[Title/Abstract])) OR (small cell cancer, lung[Title/Abstract])) OR (small cell carcinoma of the lung[Title/Abstract])) OR (small cell lung tumor[Title/Abstract])) OR (small cell neuroendocrine carcinoma of the lung[Title/Abstract])) OR (small cell pulmonary cancer[Title/Abstract])) OR (small cell pulmonary carcinoma[Title/Abstract])) OR (Tracheal Neoplasms[Title/Abstract])) OR (Tracheal Neoplasm[Title/Abstract])) OR (Thoracic Neoplasm[Title/Abstract])) OR (Thoracic Neoplasms[Title/Abstract])) OR (tumor of the lung[Title/Abstract])) OR (tumor, lung[Title/Abstract])) OR (tumorigenesis in the lung[Title/Abstract])) OR (tumour, lung[Title/Abstract])) OR (thoracic cancer[Title/Abstract])) OR (thoracic carcinoma[Title/Abstract])) OR (thoracic malignancies[Title/Abstract])) OR (thoracic malignancy[Title/Abstract])) OR (thoracic malignant neoplasm[Title/Abstract])) OR (thoracic malignant tumor[Title/Abstract])) OR (thoracic sarcoma[Title/Abstract])) OR (thorax cancer[Title/Abstract])

#3 #1 OR #2

#4

((((((((Deep Learning[MeSH Terms]) OR (Machine Learning[MeSH Terms])) OR (Algorithms[MeSH Terms])) OR (Artificial Intelligence[MeSH Terms])) OR (Supervised Machine Learning[MeSH Terms])) OR (Support Vector Machine[MeSH Terms])) OR (Computing Methodologies[MeSH Terms])) OR (Unsupervised Machine Learning[MeSH Terms])) OR (Computer Neural Network[MeSH Terms])

#5

((((((((((((((((((((((((((((((((((((((((((((((((((((((((((((((((((((((((((((((((((((((((((((((((((((((((((((((((((((((((((((((((((((((((((((((((((((((((((((((((Algorithms[Title/Abstract]) OR (Algorithm[Title/Abstract])) OR (Artificial Intelligence[Title/Abstract])) OR (AI (Artificial Intelligence[Title/Abstract]))) OR (AI[Title/Abstract])) OR (Acquisition, Knowledge (Computer[Title/Abstract]))) OR (Active Machine Learning[Title/Abstract])) OR (Automation[Title/Abstract])) OR (Automations[Title/Abstract])) OR (artificial neural network[Title/Abstract])) OR (artificial neural networks[Title/Abstract])) OR (algorithmic neural network[Title/Abstract])) OR (ANN (artificial neural network[Title/Abstract]))) OR (ANN analysis[Title/Abstract])) OR (ANN approach[Title/Abstract])) OR (ANN method[Title/Abstract])) OR (ANN methodology[Title/Abstract])) OR (ANN methods[Title/Abstract])) OR (ANN model[Title/Abstract])) OR (ANN models[Title/Abstract])) OR (ANN modeling[Title/Abstract])) OR (ANN modelling[Title/Abstract])) OR (ANN output[Title/Abstract])) OR (ANN technique[Title/Abstract])) OR (ANN techniques[Title/Abstract])) OR (ANN training[Title/Abstract])) OR (ANN training ANNs (artificial neural networks[Title/Abstract]))) OR (artificial NN[Title/Abstract])) OR (artificial NNs[Title/Abstract])) OR (Computer Neural Network[Title/Abstract])) OR (Computer Neural Networks[Title/Abstract])) OR (Computer Reasoning[Title/Abstract])) OR (Computer Vision Systems[Title/Abstract])) OR (Computer Vision System[Title/Abstract])) OR (Computational Neural Networks[Title/Abstract])) OR (Computational Neural Network[Title/Abstract])) OR (computerized neural network[Title/Abstract])) OR (Computing Methodologies[Title/Abstract])) OR (Computing Methodology[Title/Abstract])) OR (Computing, High Performance[Title/Abstract])) OR (Connectionist Model[Title/Abstract])) OR (Computational Intelligence[Title/Abstract])) OR (Connectionist Models[Title/Abstract])) OR (connectionist network[Title/Abstract])) OR (connectionist neural network[Title/Abstract])) OR (connectionist system[Title/Abstract])) OR (convolutional neural network[Title/Abstract])) OR (CNN (convolutional neural network[Title/Abstract]))) OR (CNNs (convolutional neural networks[Title/Abstract]))) OR (ConvNet[Title/Abstract])) OR (convoluted neural network[Title/Abstract])) OR (convolution neural network[Title/Abstract])) OR (convolutional ANNs[Title/Abstract])) OR (convolutional artificial neural network[Title/Abstract])) OR (convolutional deep neural network[Title/Abstract])) OR (convolutional NN[Title/Abstract])) OR (convolutionary neural network[Title/Abstract])) OR (convolution algorithm[Title/Abstract])) OR (convolutional algorithm[Title/Abstract])) OR (convolutionary algorithm[Title/Abstract])) OR (convolution-superposition algorithm[Title/Abstract])) OR (convolution/superposition algorithm[Title/Abstract])) OR (convolutionsuperposition algorithm[Title/Abstract])) OR (U-Net[Title/Abstract])) OR (V-Net[Title/Abstract])) OR (CNN[Title/Abstract])) OR (Deep Learning[Title/Abstract])) OR (DNN (deep neural network[Title/Abstract]))) OR (DNNs (deep neural networks[Title/Abstract]))) OR (deep convolutional neural network[Title/Abstract])) OR (deep neural network[Title/Abstract])) OR (deep artificial neural network[Title/Abstract])) OR (deep NNs[Title/Abstract])) OR (feature-based learning[Title/Abstract])) OR (feature learning (machine learning[Title/Abstract]))) OR (feature learning (machine learning[Title/Abstract]))) OR (Hierarchical Learning[Title/Abstract])) OR (High Performance Computing[Title/Abstract])) OR (Intelligence, Artificial[Title/Abstract])) OR (Intelligence, Computational[Title/Abstract])) OR (Intelligence, Machine[Title/Abstract])) OR (Inductive Machine Learning[Title/Abstract])) OR (Knowledge Acquisition (Computer[Title/Abstract]))) OR (Knowledge Representation (Computer[Title/Abstract]))) OR (Knowledge Representations (Computer[Title/Abstract]))) OR (Learning, Deep[Title/Abstract])) OR (Learning, Hierarchical[Title/Abstract])) OR (Learning, Machine[Title/Abstract])) OR (learning machines[Title/Abstract])) OR (Learning, Active Machine[Title/Abstract])) OR (Learning, Supervised Machine[Title/Abstract])) OR (Learning, Unsupervised Machine[Title/Abstract])) OR (Learning, Semi-supervised[Title/Abstract])) OR (Learning from Labeled Data[Title/Abstract])) OR (Learning, Transfer[Title/Abstract])) OR (Learning, Inductive Machine[Title/Abstract])) OR (Methodologies, Computing[Title/Abstract])) OR (Methodology, Computing[Title/Abstract])) OR (Machine Intelligence[Title/Abstract])) OR (Machine Learning[Title/Abstract])) OR (Machine Learning, Supervised[Title/Abstract])) OR (Machine Learning, Unsupervised[Title/Abstract])) OR (Machine Learning, Inductive[Title/Abstract])) OR (Machine, Support Vector[Title/Abstract])) OR (Machines, Support Vector[Title/Abstract])) OR (Models, Neural Network[Title/Abstract])) OR (Model, Neural Network[Title/Abstract])) OR (Model, Connectionist[Title/Abstract])) OR (Models, Connectionist[Title/Abstract])) OR (Machine Learning, Active[Title/Abstract])) OR (Machine Learning with a Teacher[Title/Abstract])) OR (mathematical neural network[Title/Abstract])) OR (manifold learning [+NT])) OR (Network, Support Vector[Title/Abstract])) OR (Networks, Support Vector[Title/Abstract])) OR (Network, Computer Neural[Title/Abstract])) OR (Networks, Computer Neural[Title/Abstract])) OR (Network, Neural (Computer[Title/Abstract]))) OR (Network Model, Neural[Title/Abstract])) OR (Network Models, Neural[Title/Abstract])) OR (Networks, Neural (Computer[Title/Abstract]))) OR (Network, Computational Neural[Title/Abstract])) OR (Networks, Computational Neural[Title/Abstract])) OR (Neural Network, Computer[Title/Abstract])) OR (Neural Network Model[Title/Abstract])) OR (Neural Network Models[Title/Abstract])) OR (Neural Network, Computational[Title/Abstract])) OR (Neural Networks, Computational[Title/Abstract])) OR (Neural Networks, Computer[Title/Abstract])) OR (Neural Network (Computer[Title/Abstract]))) OR (Neural Networks (Computer[Title/Abstract]))) OR (Neural Network (Computer)Neural Networks, Computer[Title/Abstract])) OR (neural network (artificial[Title/Abstract]))) OR (neural network algorithm[Title/Abstract])) OR (Perceptrons[Title/Abstract])) OR (Perceptron[Title/Abstract])) OR (Performance Computing, High[Title/Abstract])) OR (Representation, Knowledge (Computer[Title/Abstract]))) OR (Reasoning, Computer[Title/Abstract])) OR (representation learning[Title/Abstract])) OR (representation-based learning[Title/Abstract])) OR (System, Computer Vision[Title/Abstract])) OR (Systems, Computer Vision[Title/Abstract])) OR (Semi-supervised Learning[Title/Abstract])) OR (Semi supervised Learning[Title/Abstract])) OR (Supervised Machine Learning[Title/Abstract])) OR (Support Vector Machine[Title/Abstract])) OR (Support Vector Machines[Title/Abstract])) OR (Support Vector Network[Title/Abstract])) OR (Support Vector Networks[Title/Abstract])) OR (superposition-convolution algorithm[Title/Abstract])) OR (superposition/convolution algorithm[Title/Abstract])) OR (superpositionconvolution algorithm[Title/Abstract])) OR (Transfer Learning[Title/Abstract])) OR (Unsupervised Machine Learning[Title/Abstract])) OR (Vision System, Computer[Title/Abstract])) OR (Vision Systems, Computer[Title/Abstract])) OR (Vector Machine, Support[Title/Abstract])) OR (Vector Machines, Support[Title/Abstract])) OR (Vector Network, Support[Title/Abstract])) OR (Vector Networks, Support[Title/Abstract])

#6 #4 OR #5

#7

(Segment[Title/Abstract]) OR (segmentation[Title/Abstract])

#8 #3 AND #6

#9 #8 AND #7

**2.** **Embase (n = 2361 )**

#10. #3 AND #6 AND #9

#9. #7 OR #8

#8. 'region growing':ti,ab,kw AND imaging:ti,ab,kw OR

'segmentation algorithm':ti,ab,kw OR 'image

segmentation algorithm':ti,ab,kw OR 'image

segmentation':ti,ab,kw OR (imaging:ti,ab,kw AND

display:ti,ab,kw) OR 'imaging algorithm':ti,ab,kw

OR 'image algorithm':ti,ab,kw OR 'image analysis

algorithm':ti,ab,kw OR 'image enhancement

algorithm':ti,ab,kw OR 'image processing

algorithm':ti,ab,kw OR 'imaging processing

algorithm':ti,ab,kw OR 'segmentation':ti,ab,kw OR

'segment':ti,ab,kw

#7. 'image segmentation'/exp OR 'segmentation

algorithm'/exp OR 'region growing (imaging)'/exp

OR 'imaging algorithm'/exp

#6. #4 OR #5

#5. 'algorithms':ti,ab,kw OR 'algorithm':ti,ab,kw OR

'artificial intelligence':ti,ab,kw OR

(ai:ti,ab,kw AND 'artificial

intelligence':ti,ab,kw) OR ('acquisition,

knowledge':ti,ab,kw AND computer:ti,ab,kw) OR

'active machine learning':ti,ab,kw OR

'automation':ti,ab,kw OR 'automations':ti,ab,kw

OR 'artificial neural network':ti,ab,kw OR

'artificial neural networks':ti,ab,kw OR

'algorithmic neural network':ti,ab,kw OR

(ann:ti,ab,kw AND 'artificial neural

network':ti,ab,kw) OR 'ann analysis':ti,ab,kw OR

'ann approach':ti,ab,kw OR 'ann method':ti,ab,kw

OR 'ann methodology':ti,ab,kw OR 'ann

methods':ti,ab,kw OR 'ann model':ti,ab,kw OR 'ann

models':ti,ab,kw OR 'ann modeling':ti,ab,kw OR

'ann modelling':ti,ab,kw OR 'ann output':ti,ab,kw

OR 'ann technique':ti,ab,kw OR 'ann

techniques':ti,ab,kw OR 'ann training':ti,ab,kw

OR (anns:ti,ab,kw AND 'artificial neural

networks':ti,ab,kw) OR 'artificial nn':ti,ab,kw

OR 'artificial nns':ti,ab,kw OR 'computer neural

network':ti,ab,kw OR 'computer neural

networks':ti,ab,kw OR 'computer

reasoning':ti,ab,kw OR 'computer vision

systems':ti,ab,kw OR 'computer vision

system':ti,ab,kw OR 'computational neural

networks':ti,ab,kw OR 'computational neural

network':ti,ab,kw OR 'computerized neural

network':ti,ab,kw OR 'computing

methodologies':ti,ab,kw OR 'computing

methodology':ti,ab,kw OR 'computing, high

performance':ti,ab,kw OR 'connectionist

model':ti,ab,kw OR 'computational

intelligence':ti,ab,kw OR 'connectionist

models':ti,ab,kw OR 'connectionist

network':ti,ab,kw OR 'connectionist neural

network':ti,ab,kw OR 'connectionist

system':ti,ab,kw OR 'convolutional neural

network':ti,ab,kw OR (cnn:ti,ab,kw AND

'convolutional neural network':ti,ab,kw) OR

(cnns:ti,ab,kw AND 'convolutional neural

networks':ti,ab,kw) OR 'convnet':ti,ab,kw OR

'convoluted neural network':ti,ab,kw OR

'convolution neural network':ti,ab,kw OR

'convolutional anns':ti,ab,kw OR 'convolutional

artificial neural network':ti,ab,kw OR

'convolutional deep neural network':ti,ab,kw OR

'convolutional nn':ti,ab,kw OR 'convolutionary

neural network':ti,ab,kw OR 'convolution

algorithm':ti,ab,kw OR 'convolutional

algorithm':ti,ab,kw OR 'convolutionary

algorithm':ti,ab,kw OR 'convolution-superposition

algorithm':ti,ab,kw OR 'convolution/superposition

algorithm':ti,ab,kw OR 'convolutionsuperposition

algorithm':ti,ab,kw OR 'u-net':ti,ab,kw OR

'v-net':ti,ab,kw OR 'cnn':ti,ab,kw OR 'deep

learning':ti,ab,kw OR (dnn:ti,ab,kw AND 'deep

neural network':ti,ab,kw) OR (dnns:ti,ab,kw AND

'deep neural networks':ti,ab,kw) OR 'deep

convolutional neural network':ti,ab,kw OR 'deep

neural network':ti,ab,kw OR 'deep artificial

neural network':ti,ab,kw OR 'deep nns':ti,ab,kw

OR 'feature-based learning':ti,ab,kw OR ('feature

learning':ti,ab,kw AND 'machine

learning':ti,ab,kw) OR 'hierarchical

learning':ti,ab,kw OR 'high performance

computing':ti,ab,kw OR 'intelligence,

artificial':ti,ab,kw OR 'intelligence,

computational':ti,ab,kw OR 'intelligence,

machine':ti,ab,kw OR 'inductive machine

learning':ti,ab,kw OR ('knowledge

acquisition':ti,ab,kw AND computer:ti,ab,kw) OR

('knowledge representation':ti,ab,kw AND

computer:ti,ab,kw) OR ('knowledge

representations':ti,ab,kw AND computer:ti,ab,kw)

OR 'learning, deep':ti,ab,kw OR 'learning,

hierarchical':ti,ab,kw OR 'learning,

machine':ti,ab,kw OR 'learning machines':ti,ab,kw

OR 'learning, active machine':ti,ab,kw OR

'learning, supervised machine':ti,ab,kw OR

'learning, unsupervised machine':ti,ab,kw OR

'learning, semi-supervised':ti,ab,kw OR 'learning

from labeled data':ti,ab,kw OR 'learning,

transfer':ti,ab,kw OR 'learning, inductive

machine':ti,ab,kw OR 'methodologies,

computing':ti,ab,kw OR 'methodology,

computing':ti,ab,kw OR 'machine

intelligence':ti,ab,kw OR 'machine

learning':ti,ab,kw OR 'machine learning,

supervised':ti,ab,kw OR 'machine learning,

unsupervised':ti,ab,kw OR 'machine learning,

inductive':ti,ab,kw OR 'machine, support

vector':ti,ab,kw OR 'machines, support

vector':ti,ab,kw OR 'models, neural

network':ti,ab,kw OR 'model, neural

network':ti,ab,kw OR 'model,

connectionist':ti,ab,kw OR 'models,

connectionist':ti,ab,kw OR 'machine learning,

active':ti,ab,kw OR 'machine learning with a

teacher':ti,ab,kw OR 'mathematical neural

network':ti,ab,kw OR 'manifold learning

[+nt]':ti,ab,kw OR 'network, support

vector':ti,ab,kw OR 'networks, support

vector':ti,ab,kw OR 'network, computer

neural':ti,ab,kw OR 'networks, computer

neural':ti,ab,kw OR ('network, neural':ti,ab,kw

AND computer:ti,ab,kw) OR 'network model,

neural':ti,ab,kw OR 'network models,

neural':ti,ab,kw OR ('networks, neural':ti,ab,kw

AND computer:ti,ab,kw) OR 'network, computational

neural':ti,ab,kw OR 'networks, computational

neural':ti,ab,kw OR 'neural network,

computer':ti,ab,kw OR 'neural network

model':ti,ab,kw OR 'neural network

models':ti,ab,kw OR 'neural network,

computational':ti,ab,kw OR 'neural networks,

computational':ti,ab,kw OR 'neural networks,

computer':ti,ab,kw OR ('neural network':ti,ab,kw

AND computer:ti,ab,kw) OR ('neural

networks':ti,ab,kw AND computer:ti,ab,kw) OR

('neural network':ti,ab,kw AND computer:ti,ab,kw

AND 'neural networks, computer':ti,ab,kw) OR

('neural network':ti,ab,kw AND

artificial:ti,ab,kw) OR 'neural network

algorithm':ti,ab,kw OR 'perceptrons':ti,ab,kw OR

'perceptron':ti,ab,kw OR 'performance computing,

high':ti,ab,kw OR ('representation,

knowledge':ti,ab,kw AND computer:ti,ab,kw) OR

'reasoning, computer':ti,ab,kw OR 'representation

learning':ti,ab,kw OR 'representation-based

learning':ti,ab,kw OR 'system, computer

vision':ti,ab,kw OR 'systems, computer

vision':ti,ab,kw OR 'semi-supervised

learning':ti,ab,kw OR 'semi supervised

learning':ti,ab,kw OR 'supervised machine

learning':ti,ab,kw OR 'support vector

machine':ti,ab,kw OR 'support vector

machines':ti,ab,kw OR 'support vector

network':ti,ab,kw OR 'support vector

networks':ti,ab,kw OR 'superposition-convolution

algorithm':ti,ab,kw OR 'superposition/convolution

algorithm':ti,ab,kw OR 'superpositionconvolution

algorithm':ti,ab,kw OR 'transfer

learning':ti,ab,kw OR 'unsupervised machine

learning':ti,ab,kw OR 'vision system,

computer':ti,ab,kw OR 'vision systems,

computer':ti,ab,kw OR 'vector machine,

support':ti,ab,kw OR 'vector machines,

support':ti,ab,kw OR 'vector network,

support':ti,ab,kw OR 'vector networks,

support':ti,ab,kw

#4. 'deep learning'/exp OR 'feature learning (machine

learning)'/exp OR 'machine learning'/exp OR

'artificial neural network'/exp OR 'deep neural

network'/exp OR 'convolutional neural

network'/exp OR 'convolution algorithm'/exp OR

'artificial intelligence'/exp OR

'convolution-superposition algorithm'/exp

#3. #1 OR #2

#2. 'adenocarcinoma, lung':ti,ab,kw OR

'adenocarcinomas, lung':ti,ab,kw OR

'adenocarcinoma, bronchiolo-alveolar':ti,ab,kw OR

'adenocarcinoma, bronchiolo alveolar':ti,ab,kw OR

'adenocarcinomas, bronchiolo-alveolar':ti,ab,kw

OR 'adenocarcinoma, alveolar':ti,ab,kw OR

'adenocarcinomas, alveolar':ti,ab,kw OR

'adenocarcinoma of lung':ti,ab,kw OR 'alveolar

adenocarcinoma':ti,ab,kw OR 'alveolar

adenocarcinomas':ti,ab,kw OR 'alveolar

carcinoma':ti,ab,kw OR 'alveolar

carcinomas':ti,ab,kw OR 'alveolar cell

carcinoma':ti,ab,kw OR 'alveolar cell

carcinomas':ti,ab,kw OR 'alveobronchial

carcinoma':ti,ab,kw OR 'alveolobronchiolar

carcinoma':ti,ab,kw OR 'alveolar cell

cancer':ti,ab,kw OR 'alveolar clear cell

sarcoma':ti,ab,kw OR 'alveolar lung

carcinoma':ti,ab,kw OR 'alveolus cell

cancer':ti,ab,kw OR 'alveolus cell cancer,

lung':ti,ab,kw OR 'bronchiolo-alveolar

carcinoma':ti,ab,kw OR 'bronchiolo-alveolar

carcinomas':ti,ab,kw OR 'bronchiolo-alveolar

adenocarcinoma':ti,ab,kw OR 'bronchiolo-alveolar

adenocarcinomas':ti,ab,kw OR 'bronchial

carcinoma':ti,ab,kw OR 'bronchial

carcinomas':ti,ab,kw OR 'bronchogenic

carcinoma':ti,ab,kw OR 'bronchogenic

carcinomas':ti,ab,kw OR 'bronchiolar

carcinoma':ti,ab,kw OR 'bronchiolar

carcinomas':ti,ab,kw OR 'bronchioloalveolar

carcinomas':ti,ab,kw OR 'bronchial

neoplasm':ti,ab,kw OR 'bronchial

neoplasms':ti,ab,kw OR 'bronchioloalveolar cell

carcinoma':ti,ab,kw OR 'bronchioloalveolar

carcinoma':ti,ab,kw OR 'broncho alveolar

carcinoma':ti,ab,kw OR 'bronchioalveolar lung

carcinoma':ti,ab,kw OR 'bronchiolar

cancer':ti,ab,kw OR 'bronchiolar cell

cancer':ti,ab,kw OR 'bronchiolar cell

carcinoma':ti,ab,kw OR 'bronchoalveolar

carcinoma':ti,ab,kw OR 'bronchoalveolar

cancer':ti,ab,kw OR 'bronchial

carcinomata':ti,ab,kw OR 'broncho-pulmonary

carcinoma':ti,ab,kw OR 'bronchogenic carcinoma of

the lung':ti,ab,kw OR 'bronchogenic lung

carcinoma':ti,ab,kw OR 'bronchopulmonary

carcinoma':ti,ab,kw OR 'bronchus

carcinomacarcinoma, bronchiolar':ti,ab,kw OR

'bronchopulmonary cancer':ti,ab,kw OR

'broncho-pulmonary neoplasm':ti,ab,kw OR

'broncho-pulmonary tumor':ti,ab,kw OR

'bronchopulmonary neoplasia':ti,ab,kw OR

'bronchopulmonary neoplasm':ti,ab,kw OR

'bronchopulmonary tumorca lung':ti,ab,kw OR

'bronchial small cell cancer':ti,ab,kw OR

'bronchial small cell carcinoma':ti,ab,kw OR

'bronchial non small cell cancer':ti,ab,kw OR

'bronchial non small cell carcinoma':ti,ab,kw OR

'cancer, lung':ti,ab,kw OR 'cancers,

lung':ti,ab,kw OR 'cancer, pulmonary':ti,ab,kw OR

'cancers, pulmonary':ti,ab,kw OR 'cancer of the

lung':ti,ab,kw OR 'cancer of lung':ti,ab,kw OR

'cancer of the respiratory tract':ti,ab,kw OR

'cancer in the thorax':ti,ab,kw OR 'cancer of the

thoracic cavity':ti,ab,kw OR 'cancer of the

thorax':ti,ab,kw OR ('cancer of the

thorax':ti,ab,kw AND 'thoracic cavity':ti,ab,kw)

OR 'cancerous lung cell line':ti,ab,kw OR

'cancerous pulmonary cell line':ti,ab,kw OR

'carcinoma, alveolar':ti,ab,kw OR 'carcinomas,

alveolar':ti,ab,kw OR 'carcinoma, alveolar

cell':ti,ab,kw OR 'carcinomas, alveolar

cell':ti,ab,kw OR 'carcinoma, bronchial':ti,ab,kw

OR 'carcinomas, bronchial':ti,ab,kw OR

'carcinoma, bronchiolar':ti,ab,kw OR 'carcinomas,

bronchiolar':ti,ab,kw OR 'carcinoma,

bronchogenic':ti,ab,kw OR 'carcinomas,

bronchogenic':ti,ab,kw OR 'carcinoma,

bronchiolo-alveolar':ti,ab,kw OR 'carcinoma,

bronchiolo alveolar':ti,ab,kw OR 'carcinomas,

bronchiolo-alveolar':ti,ab,kw OR 'carcinoma,

bronchioloalveolar':ti,ab,kw OR 'carcinomas,

bronchioloalveolar':ti,ab,kw OR 'carcinoma,

non-small-cell lung':ti,ab,kw OR 'carcinoma, non

small cell lung':ti,ab,kw OR 'carcinomas,

non-small-cell lung':ti,ab,kw OR 'carcinoma,

non-small cell lung':ti,ab,kw OR 'carcinoma, oat

cell':ti,ab,kw OR 'carcinomas, oat cell':ti,ab,kw

OR 'carcinoma, small cell lung':ti,ab,kw OR

'carcinoma, small cell':ti,ab,kw OR 'carcinomas,

small cell':ti,ab,kw OR 'carcinoma of the

thorax':ti,ab,kw OR 'carcinoma of lung':ti,ab,kw

OR 'carcinoma of the lung':ti,ab,kw OR 'carcinoma

of the bronchus':ti,ab,kw OR 'carcinoma of

bronchus':ti,ab,kw OR 'carcinoma, lewis':ti,ab,kw

OR 'carcinoma, lewis lung':ti,ab,kw OR

'carcinoma, lung':ti,ab,kw OR 'carcinoma

pulmonum':ti,ab,kw OR 'carcinomatosis of the

lung':ti,ab,kw OR 'carcinogenesis in the

respiratory tract':ti,ab,kw OR 'carcinogenesis of

the lungbroncho-pulmonary cancer':ti,ab,kw OR

'carcinomatous lung':ti,ab,kw OR 'carcinomatous

pulmonary':ti,ab,kw OR 'invasive lung

carcinoma':ti,ab,kw OR 'invasive pulmonary

carcinoma':ti,ab,kw OR 'lung

adenocarcinoma':ti,ab,kw OR 'lung

adenocarcinomas':ti,ab,kw OR 'lung

cancer':ti,ab,kw OR 'lung cancers':ti,ab,kw OR

'lung carcinoma, non-small-cell':ti,ab,kw OR

'lung carcinomas, non-small-cell':ti,ab,kw OR

'lung neoplasm':ti,ab,kw OR 'lung

neoplasms':ti,ab,kw OR 'lung nodule,

solitary':ti,ab,kw OR 'lung alveolar

carcinoma':ti,ab,kw OR 'lung alveolus cell

carcinoma':ti,ab,kw OR 'lung alveolar cell

cancer':ti,ab,kw OR 'lung alveolar cell

carcinoma':ti,ab,kw OR 'lung alveolus cell

cancer':ti,ab,kw OR 'lung cancer cell

line':ti,ab,kw OR 'lung cancer-derived cell

line':ti,ab,kw OR 'lung cancerous cell

line':ti,ab,kw OR 'lung cancers cell

line':ti,ab,kw OR 'lung carcinoma':ti,ab,kw OR

'lung carcinomata':ti,ab,kw OR 'lung

carcinomatosis':ti,ab,kw OR 'lung cavitary

carcinoma':ti,ab,kw OR 'lung

carcinogenesis':ti,ab,kw OR 'lung

cancerogenesismicrocellular lung

carcinoma':ti,ab,kw OR 'lung cancermalignancies

of the lung':ti,ab,kw OR 'lung cancer, non small

cell':ti,ab,kw OR 'lung malignancy':ti,ab,kw OR

'lung malignancies':ti,ab,kw OR 'lung

neoplasia':ti,ab,kw OR 'lung non small cell

cancer':ti,ab,kw OR 'lung non small cell

carcinoma':ti,ab,kw OR 'lung oat cell

carcinoma':ti,ab,kw OR 'lung small cell

carcinoma':ti,ab,kw OR 'lung small cell

cancer':ti,ab,kw OR 'lung tumor':ti,ab,kw OR

'lung tumour':ti,ab,kw OR 'lung

tumorigenesis':ti,ab,kw OR 'lewis lung

carcinoma':ti,ab,kw OR 'lewis lung

tumor':ti,ab,kw OR 'lewis lung tumour':ti,ab,kw

OR 'multiple pulmonary nodule':ti,ab,kw OR

'multiple pulmonary nodules':ti,ab,kw OR

'malignancy of the lung':ti,ab,kw OR 'malignant

lung neoplasm':ti,ab,kw OR 'malignant lung

tumor':ti,ab,kw OR 'malignant neoplasm of the

lung':ti,ab,kw OR 'malignant tumor of the

lung':ti,ab,kw OR 'malignancies of the

respiratory tract':ti,ab,kw OR 'malignant lung

cell line':ti,ab,kw OR 'malignant pulmonary cell

line':ti,ab,kw OR 'neoplasm, bronchial':ti,ab,kw

OR 'neoplasms, bronchial':ti,ab,kw OR 'neoplasm,

lung':ti,ab,kw OR 'neoplasms, lung':ti,ab,kw OR

'neoplasm, pulmonary':ti,ab,kw OR 'neoplasms,

pulmonary':ti,ab,kw OR 'neoplasm, respiratory

tract':ti,ab,kw OR 'neoplasm, respiratory

system':ti,ab,kw OR 'neoplasm,

respiratory':ti,ab,kw OR 'neoplasm,

tracheal':ti,ab,kw OR 'neoplasms,

tracheal':ti,ab,kw OR 'neoplasm,

thoracic':ti,ab,kw OR 'neoplasms,

thoracic':ti,ab,kw OR 'non small cell lung

cancer':ti,ab,kw OR 'non-small cell lung

cancer':ti,ab,kw OR 'non small cell lung

carcinoma':ti,ab,kw OR 'non-small-cell lung

carcinomas':ti,ab,kw OR 'non-small-cell lung

carcinoma':ti,ab,kw OR 'non-small cell lung

carcinoma':ti,ab,kw OR 'nonsmall cell lung

cancer':ti,ab,kw OR 'non oat cell lung

cancer':ti,ab,kw OR 'non small cell bronchial

cancer':ti,ab,kw OR 'non small cell cancer,

lung':ti,ab,kw OR 'non small cell pulmonary

carcinoma':ti,ab,kw OR 'non squamous

nsclc':ti,ab,kw OR 'nonsmall cell carcinoma of

the lung':ti,ab,kw OR 'nonsmall cell lung

carcinoma':ti,ab,kw OR 'nodule, solitary

lung':ti,ab,kw OR 'nodule, solitary

pulmonary':ti,ab,kw OR 'nodules, solitary

pulmonary':ti,ab,kw OR 'neoplasia of the

lung':ti,ab,kw OR 'neoplastic lung':ti,ab,kw OR

'oat cell carcinoma of lung':ti,ab,kw OR 'oat

cell lung cancer':ti,ab,kw OR 'oat cell

carcinoma':ti,ab,kw OR 'oat cell

carcinomas':ti,ab,kw OR 'oat cell cancer of the

lung':ti,ab,kw OR 'oat cell carcinoma of the

lung':ti,ab,kw OR 'oat cell lung

carcinoma':ti,ab,kw OR 'pulmonary

cancer':ti,ab,kw OR 'pulmonary cancers':ti,ab,kw

OR 'pulmonary neoplasm':ti,ab,kw OR 'pulmonary

neoplasms':ti,ab,kw OR 'pulmonary nodule,

solitary':ti,ab,kw OR 'pulmonary nodules,

solitary':ti,ab,kw OR 'pulmonary nodule,

multiple':ti,ab,kw OR 'pulmonary nodules,

multiple':ti,ab,kw OR 'pulmonary alveolar cell

cancer':ti,ab,kw OR 'pulmonary alveolus cell

cancer':ti,ab,kw OR 'pulmonary cancer cell

line':ti,ab,kw OR 'pulmonary

cancerogenesis':ti,ab,kw OR 'pulmonary

carcinogenesis':ti,ab,kw OR 'peribronchial

carcinoma':ti,ab,kw OR 'pulmonary

carcinoma':ti,ab,kw OR 'pulmonary

carcinomatosis':ti,ab,kw OR 'pulmonary

malignancies':ti,ab,kw OR 'pulmonary

malignancy':ti,ab,kw OR 'pulmonary

neoplasia':ti,ab,kw OR 'pulmonary non small cell

cancer':ti,ab,kw OR 'pulmonary non small cell

carcinoma':ti,ab,kw OR 'pulmonary small cell

cancer':ti,ab,kw OR 'pulmonary small cell

carcinomapulmonary tumor':ti,ab,kw OR 'pulmonary

tumorigenesis':ti,ab,kw OR 'pulmonary

tumour':ti,ab,kw OR 'respiratory tract

neoplasms':ti,ab,kw OR 'respiratory tract

neoplasm':ti,ab,kw OR 'respiratory system

neoplasms':ti,ab,kw OR 'respiratory system

neoplasm':ti,ab,kw OR 'respiratory

neoplasms':ti,ab,kw OR 'respiratory

neoplasm':ti,ab,kw OR 'respiratory tract

cancer':ti,ab,kw OR 'respiratory cancer':ti,ab,kw

OR 'respiratory carcinogenesis':ti,ab,kw OR

'respiratory malignancy':ti,ab,kw OR 'respiratory

malignancies':ti,ab,kw OR 'respiratory tract

carcinogenesis':ti,ab,kw OR 'respiratory tract

malignancies':ti,ab,kw OR 'respiratory tract

malignancy':ti,ab,kw OR 'respiratory tract

carcinoma':ti,ab,kw OR 'respiratory system

carcinoma':ti,ab,kw OR 'small cell lung

cancer':ti,ab,kw OR 'small cell cancer of the

lung':ti,ab,kw OR 'small cell carcinoma':ti,ab,kw

OR 'small cell carcinomas':ti,ab,kw OR 'small

cell lung carcinoma':ti,ab,kw OR 'solitary

pulmonary nodule':ti,ab,kw OR 'solitary pulmonary

nodules':ti,ab,kw OR 'solitary lung

nodule':ti,ab,kw OR 'solitary lung

nodules':ti,ab,kw OR 'small cell bronchial

cancer':ti,ab,kw OR 'small cell bronchial

carcinoma':ti,ab,kw OR 'small cell cancer,

lung':ti,ab,kw OR 'small cell carcinoma of the

lung':ti,ab,kw OR 'small cell lung

tumor':ti,ab,kw OR 'small cell neuroendocrine

carcinoma of the lung':ti,ab,kw OR 'small cell

pulmonary cancer':ti,ab,kw OR 'small cell

pulmonary carcinoma':ti,ab,kw OR 'tracheal

neoplasms':ti,ab,kw OR 'tracheal

neoplasm':ti,ab,kw OR 'thoracic

neoplasm':ti,ab,kw OR 'thoracic

neoplasms':ti,ab,kw OR 'tumor of the

lung':ti,ab,kw OR 'tumor, lung':ti,ab,kw OR

'tumorigenesis in the lung':ti,ab,kw OR 'tumour,

lung':ti,ab,kw OR 'thoracic cancer':ti,ab,kw OR

'thoracic carcinoma':ti,ab,kw OR 'thoracic

malignancies':ti,ab,kw OR 'thoracic

malignancy':ti,ab,kw OR 'thoracic malignant

neoplasm':ti,ab,kw OR 'thoracic malignant

tumor':ti,ab,kw OR 'thoracic sarcoma':ti,ab,kw OR

'thorax cancer':ti,ab,kw

#1. 'lung cancer'/exp OR 'bronchus cancer'/exp OR

'lung tumor'/exp OR 'respiratory tract tumor'/exp

OR 'lung nodule'/exp OR 'lung carcinoma'/exp OR

'invasive lung carcinoma'/exp OR 'lewis

carcinoma'/exp OR 'lung alveolus cell

carcinoma'/exp OR 'non small cell lung

cancer'/exp OR 'large cell lung carcinoma'/exp OR

'large cell neuroendocrine carcinoma'/exp OR

'trachea cancer'/exp OR 'small cell lung

cancer'/exp OR 'lung adenocarcinoma'/exp OR

'squamous cell lung carcinoma'/exp OR 'lung

carcinogenesis'/exp OR 'thoracic cancer'/exp OR

'respiratory tract carcinoma'/exp OR 'respiratory

tract cancer'/exp OR 'lung cancer cell line'/exp

OR 'lung tumor cell line'/exp

**3. Web of Science Core Collection search strategy (n = 3173)**

#1 TS=(Adenocarcinoma, Lung OR Adenocarcinomas, Lung OR Adenocarcinoma, Bronchiolo-Alveolar OR Adenocarcinoma, Bronchiolo Alveolar OR Adenocarcinomas, Bronchiolo-Alveolar OR Adenocarcinoma, Alveolar OR Adenocarcinomas, Alveolar OR Adenocarcinoma of Lung OR Alveolar Adenocarcinoma OR Alveolar Adenocarcinomas OR Alveolar Carcinoma OR Alveolar Carcinomas OR Alveolar Cell Carcinoma OR Alveolar Cell Carcinomas OR alveobronchial carcinoma OR alveolobronchiolar carcinoma OR alveolar cell cancer OR alveolar clear cell sarcoma OR alveolar lung carcinoma OR alveolus cell cancer OR alveolus cell cancer, lung OR Bronchiolo-Alveolar Carcinoma OR Bronchiolo-Alveolar Carcinomas OR Bronchiolo-Alveolar Adenocarcinoma OR Bronchiolo-Alveolar Adenocarcinomas OR Bronchial Carcinoma OR Bronchial Carcinomas OR Bronchogenic Carcinoma OR Bronchogenic Carcinomas OR Bronchiolar Carcinoma OR Bronchiolar Carcinomas OR Bronchioloalveolar Carcinoma OR Bronchioloalveolar Carcinomas OR Bronchial Neoplasm OR Bronchial Neoplasms OR bronchioloalveolar cell carcinoma OR bronchioloalveolar carcinoma OR broncho alveolar carcinoma OR bronchioalveolar lung carcinoma OR bronchiolar cancer OR bronchiolar cell cancer OR bronchiolar cell carcinoma OR bronchoalveolar carcinoma OR bronchoalveolar cancer OR bronchial carcinomata OR broncho-pulmonary carcinoma OR bronchogenic carcinoma of the lung OR bronchogenic lung carcinoma OR bronchopulmonary carcinoma OR bronchus carcinomacarcinoma, bronchiolar OR bronchopulmonary cancer OR broncho-pulmonary neoplasm OR broncho-pulmonary tumor OR bronchopulmonary neoplasia OR bronchopulmonary neoplasm OR bronchopulmonary tumorCa lung OR bronchial small cell cancer OR bronchial small cell carcinoma OR bronchial non small cell cancer OR bronchial non small cell carcinoma OR Cancer, Lung OR Cancers, Lung OR Cancer, Pulmonary OR Cancers, Pulmonary OR Cancer of the Lung OR Cancer of Lung OR cancer of the respiratory tract OR cancer in the thorax OR cancer of the thoracic cavity OR cancer of the thorax OR cancer of the thorax and thoracic cavity OR cancerous lung cell line OR cancerous pulmonary cell line OR Carcinoma, Alveolar OR Carcinomas, Alveolar OR Carcinoma, Alveolar Cell OR Carcinomas, Alveolar Cell OR Carcinoma, Bronchial OR Carcinomas, Bronchial OR Carcinoma, Bronchiolar OR Carcinomas, Bronchiolar OR Carcinoma, Bronchogenic OR Carcinomas, Bronchogenic OR Carcinoma, Bronchiolo-Alveolar OR Carcinoma, Bronchiolo Alveolar OR Carcinomas, Bronchiolo-Alveolar OR Carcinoma, Bronchioloalveolar OR Carcinomas, Bronchioloalveolar OR Carcinoma, Non-Small-Cell Lung OR Carcinoma, Non Small Cell Lung OR Carcinomas, Non-Small-Cell Lung OR Carcinoma, Non-Small Cell Lung OR Carcinoma, Non-Small-Cell Lung OR Carcinoma, Non Small Cell Lung OR Carcinomas, Non-Small-Cell Lung OR Carcinoma, Non-Small Cell Lung OR Carcinoma, Oat Cell OR Carcinomas, Oat Cell OR Carcinoma, Small Cell Lung OR Carcinoma, Small Cell OR Carcinomas, Small Cell OR carcinoma of the thorax OR carcinoma of lung OR carcinoma of the lung OR carcinoma of the bronchus OR carcinoma of bronchus OR carcinoma, lewis OR carcinoma, lewis lung OR carcinoma, lung OR carcinoma pulmonum OR carcinomatosis of the lung OR carcinogenesis in the respiratory tract OR carcinogenesis of the lungbroncho-pulmonary cancer OR carcinomatous lung OR carcinomatous pulmonary OR invasive lung carcinoma OR invasive pulmonary carcinoma OR Lung Adenocarcinoma OR Lung Adenocarcinomas OR Lung Cancer OR Lung Cancers OR Lung Carcinoma, Non-Small-Cell OR Lung Carcinomas, Non-Small-Cell OR Lung Neoplasm OR Lung Neoplasms OR Lung Nodule, Solitary OR lung alveolar carcinoma OR lung alveolus cell carcinoma OR lung alveolar cell cancer OR lung alveolar cell carcinoma OR lung alveolus cell cancer OR lung cancer cell line OR lung cancer-derived cell line OR lung cancerous cell line OR lung cancers cell line OR lung carcinoma OR lung carcinomata OR lung carcinomatosis OR lung cavitary carcinoma OR lung carcinogenesis OR microcellular lung carcinoma OR lung cancermalignancies of the lung OR lung cancer, non small cell OR lung malignancy OR lung malignancies OR lung neoplasia OR lung non small cell cancer OR lung non small cell carcinoma OR lung oat cell carcinoma OR lung small cell carcinoma OR lung small cell cancer OR lung tumor OR lung tumour OR lung tumorigenesis OR lewis lung carcinoma OR lewis lung tumor OR lewis lung tumour OR Multiple Pulmonary Nodule OR Multiple Pulmonary Nodules OR malignancy of the lung OR malignant lung neoplasm OR malignant lung tumor OR malignant neoplasm of the lung OR malignant tumor of the lung OR malignancies of the respiratory tract OR malignant lung cell line OR malignant pulmonary cell line OR Neoplasm, Bronchial OR Neoplasms, Bronchial OR Neoplasm, Lung OR Neoplasms, Lung OR Neoplasm, Pulmonary OR Neoplasms, Pulmonary OR Neoplasm, Respiratory Tract OR Neoplasm, Respiratory System OR Neoplasm, Respiratory OR Neoplasm, Tracheal OR Neoplasms, Tracheal OR Neoplasm, Thoracic OR Neoplasms, Thoracic OR non small cell lung cancer OR Non-Small Cell Lung Cancer OR Non Small Cell Lung Carcinoma OR Non-Small-Cell Lung Carcinoma OR Non-Small-Cell Lung Carcinomas OR Non-Small Cell Lung Carcinoma OR Non-Small-Cell Lung Carcinoma OR Non-Small Cell Lung Carcinoma OR Nonsmall Cell Lung Cancer OR Nonsmall Cell Lung Cancer OR non oat cell lung cancer OR non small cell bronchial cancer OR non small cell cancer, lung OR non small cell pulmonary cancer OR non small cell pulmonary carcinoma OR non squamous NSCLC OR nonsmall cell carcinoma of the lung OR nonsmall cell lung carcinoma OR Nodule, Solitary Lung OR Nodule, Solitary Pulmonary OR Nodules, Solitary Pulmonary OR neoplasia of the lung OR neoplastic lung OR Oat Cell Carcinoma of Lung OR Oat Cell Lung Cancer OR Oat Cell Carcinoma OR Oat Cell Carcinomas OR oat cell cancer of the lung OR oat cell carcinoma of the lung OR oat cell lung carcinoma OR Pulmonary Cancer OR Pulmonary Cancers OR Pulmonary Neoplasm OR Pulmonary Neoplasms OR Pulmonary Nodule, Solitary OR Pulmonary Nodules, Solitary OR Pulmonary Nodule, Multiple OR Pulmonary Nodules, Multiple OR pulmonary alveolar cell cancer OR pulmonary alveolus cell cancer OR pulmonary cancer cell line OR pulmonary cancerogenesis OR pulmonary carcinogenesis OR peribronchial carcinoma OR pulmonary carcinoma OR pulmonary carcinomatosis OR pulmonary malignancies OR pulmonary malignancy OR pulmonary neoplasia OR pulmonary non small cell cancer OR pulmonary non small cell carcinoma OR pulmonary small cell cancer OR pulmonary small cell carcinomapulmonary tumor OR pulmonary tumorigenesis OR pulmonary tumour OR Respiratory Tract Neoplasms OR Respiratory Tract Neoplasm OR Respiratory System Neoplasms OR Respiratory System Neoplasm OR Respiratory Neoplasms OR Respiratory Neoplasm OR respiratory tract cancer OR respiratory cancer OR respiratory carcinogenesis OR respiratory malignancy OR respiratory malignancies OR respiratory tract carcinogenesis OR respiratory tract malignancies OR respiratory tract malignancy OR respiratory tract carcinoma OR respiratory system carcinoma OR Small Cell Lung Cancer OR Small Cell Cancer Of The Lung OR Small Cell Carcinoma OR Small Cell Carcinomas OR Small Cell Lung Carcinoma OR Solitary Pulmonary Nodule OR Solitary Pulmonary Nodules OR Solitary Lung Nodule OR Solitary Lung Nodules OR small cell bronchial cancer OR small cell bronchial carcinoma OR small cell cancer, lung OR small cell carcinoma of the lung OR small cell lung tumor OR small cell neuroendocrine carcinoma of the lung OR small cell pulmonary cancer OR small cell pulmonary carcinoma OR Tracheal Neoplasms OR Tracheal Neoplasm OR Thoracic Neoplasm OR Thoracic Neoplasms OR tumor of the lung OR tumor, lung OR tumorigenesis in the lung OR tumour, lung OR thoracic cancer OR thoracic carcinoma OR thoracic malignancies OR thoracic malignancy OR thoracic malignant neoplasm OR thoracic malignant tumor OR thoracic sarcoma OR thorax cancer )

#2 TS=(Algorithms OR Algorithm OR Artificial Intelligence OR AI (Artificial Intelligence) OR Acquisition, Knowledge (Computer) OR Active Machine Learning OR Automation OR Automations OR artificial neural network OR artificial neural networks OR algorithmic neural network OR ANN (artificial neural network) OR ANN analysis OR ANN approach OR ANN method OR ANN methodology OR ANN methods OR ANN model OR ANN models OR ANN modeling OR ANN modelling OR ANN output OR ANN technique OR ANN techniques OR ANN training OR ANNs (artificial neural networks) OR artificial NN OR artificial NNs OR Computer Neural Network OR Computer Neural Networks OR Computer Reasoning OR Computer Vision Systems OR Computer Vision System OR Computational Neural Networks OR Computational Neural Network OR computerized neural network OR Computing Methodologies OR Computing Methodology OR Computing, High Performance OR Connectionist Model OR Computational Intelligence OR Connectionist Models OR connectionist network OR connectionist neural network OR connectionist system OR convolutional neural network OR CNN (convolutional neural network) OR CNNs (convolutional neural networks) OR ConvNet OR convoluted neural network OR convolution neural network OR convolutional ANNs OR convolutional artificial neural network OR convolutional deep neural network OR convolutional NN OR convolutionary neural network OR AI OR convolution algorithm OR convolutional algorithm OR convolutionary algorithm OR convolution-superposition algorithm OR convolution/superposition algorithm OR convolutionsuperposition algorithm OR U-Net OR V-Net OR CNN OR Deep Learning OR DNN (deep neural network) OR DNNs (deep neural networks) OR deep convolutional neural network OR deep neural network OR deep artificial neural network OR deep NNs OR feature-based learning OR feature learning (machine learning) OR feature learning (machine learning) OR Hierarchical Learning OR High Performance Computing OR Intelligence, Artificial OR Intelligence, Computational OR Intelligence, Machine OR Inductive Machine Learning OR Knowledge Acquisition (Computer) OR Knowledge Representation (Computer) OR Knowledge Representations (Computer) OR Learning, Deep OR Learning, Hierarchical OR Learning, Machine OR learning machines OR Learning, Active Machine OR Learning, Supervised Machine OR Learning, Unsupervised Machine OR Learning, Semi-supervised OR Learning from Labeled Data OR Learning, Transfer OR Learning, Inductive Machine OR Methodologies, Computing OR Methodology, Computing OR Machine Intelligence OR Machine Learning OR Machine Learning, Supervised OR Machine Learning, Unsupervised OR Machine Learning, Inductive OR Machine, Support Vector OR Machines, Support Vector OR Models, Neural Network OR Model, Neural Network OR Model, Connectionist OR Models, Connectionist OR Machine Learning, Active OR Machine Learning with a Teacher OR mathematical neural network OR manifold learning [+NT] OR Network, Support Vector OR Networks, Support Vector OR Network, Computer Neural OR Networks, Computer Neural OR Network, Neural (Computer) OR Network Model, Neural OR Network Models, Neural OR Networks, Neural (Computer) OR Network, Computational Neural OR Networks, Computational Neural OR Neural Network, Computer OR Neural Network Model OR Neural Network Models OR Neural Network, Computational OR Neural Networks, Computational OR Neural Networks, Computer OR Neural Network (Computer) OR Neural Networks (Computer) OR Neural Network (Computer)Neural Networks, Computer OR neural network (artificial) OR neural network algorithm OR Perceptrons OR Perceptron OR Performance Computing, High OR Representation, Knowledge (Computer) OR Reasoning, Computer OR representation learning OR representation-based learning OR System, Computer Vision OR Systems, Computer Vision OR Semi-supervised Learning OR Semi supervised Learning OR Supervised Machine Learning OR Support Vector Machine OR Support Vector Machines OR Support Vector Network OR Support Vector Networks OR superposition-convolution algorithm OR superposition/convolution algorithm OR superpositionconvolution algorithm OR Transfer Learning OR Unsupervised Machine Learning OR Vision System, Computer OR Vision Systems, Computer OR Vector Machine, Support OR Vector Machines, Support OR Vector Network, Support OR Vector Networks, Support)

#3 TS=(segment OR segmentation)

#4 #1 AND #2

#5 #3 AND #4

**4. Cochrane search strategy (n = 100)**

#1 MeSH descriptor: [Lung Neoplasms] explode all trees 10348

#2 MeSH descriptor: [Adenocarcinoma of Lung] explode all trees 179

#3 MeSH descriptor: [Bronchial Neoplasms] explode all trees 6466

#4 MeSH descriptor: [Multiple Pulmonary Nodules] explode all trees 91

#5 MeSH descriptor: [Adenocarcinoma, Bronchiolo-Alveolar] explode all trees 42

#6 MeSH descriptor: [Carcinoma, Bronchogenic] explode all trees 6353

#7 MeSH descriptor: [Carcinoma, Non-Small-Cell Lung] explode all trees 5734

#8 MeSH descriptor: [Small Cell Lung Carcinoma] explode all trees 534

#9 MeSH descriptor: [Thoracic Neoplasms] explode all trees 10752

#10 MeSH descriptor: [Respiratory Tract Neoplasms] explode all trees 11012

#11 MeSH descriptor: [Tracheal Neoplasms] explode all trees 9

#12 #1 OR #2 OR #3 OR #4 OR #5 OR #6 OR #7 OR #8 OR #9 OR #10 OR #11 11177

#13 (Adenocarcinoma, Lung):ti,ab,kw OR (Adenocarcinomas, Lung):ti,ab,kw OR (Adenocarcinoma, Bronchiolo-Alveolar):ti,ab,kw OR (Adenocarcinoma, Bronchiolo Alveolar):ti,ab,kw OR (Adenocarcinomas, Bronchiolo-Alveolar):ti,ab,kw (Word variations have been searched) 2457

#14 (Adenocarcinoma, Alveolar):ti,ab,kw OR (Adenocarcinomas, Alveolar):ti,ab,kw OR (Adenocarcinoma of Lung):ti,ab,kw OR (Alveolar Adenocarcinoma):ti,ab,kw OR (Alveolar Adenocarcinomas):ti,ab,kw (Word variations have been searched) 2432

#15 (Alveolar Carcinoma):ti,ab,kw OR (Alveolar Carcinomas):ti,ab,kw OR (Alveolar Cell Carcinoma):ti,ab,kw OR (Alveolar Cell Carcinomas):ti,ab,kw OR (alveobronchial carcinoma):ti,ab,kw (Word variations have been searched) 85

#16 (alveolobronchiolar carcinoma):ti,ab,kw OR (alveolar cell cancer):ti,ab,kw OR (alveolar clear cell sarcoma):ti,ab,kw OR (alveolar lung carcinoma):ti,ab,kw OR (alveolus cell cancer):ti,ab,kw (Word variations have been searched) 169

#17 (alveolus cell cancer, lung):ti,ab,kw OR (Bronchiolo-Alveolar Carcinoma):ti,ab,kw OR (Bronchiolo-Alveolar Carcinomas):ti,ab,kw OR (Bronchiolo-Alveolar Adenocarcinoma):ti,ab,kw OR (Bronchiolo-Alveolar Adenocarcinomas):ti,ab,kw (Word variations have been searched) 76

#18 (Bronchial Carcinoma):ti,ab,kw OR (Bronchial Carcinomas):ti,ab,kw OR (Bronchogenic Carcinoma):ti,ab,kw OR (Bronchogenic Carcinomas):ti,ab,kw OR (Bronchiolar Carcinoma):ti,ab,kw (Word variations have been searched) 583

#19 (Bronchiolar Carcinomas):ti,ab,kw OR (Bronchioloalveolar Carcinoma):ti,ab,kw OR (Bronchioloalveolar Carcinomas):ti,ab,kw OR (Bronchial Neoplasm):ti,ab,kw OR (Bronchial Neoplasms):ti,ab,kw (Word variations have been searched) 427

#20 (bronchioloalveolar cell carcinoma):ti,ab,kw OR (bronchioloalveolar carcinoma):ti,ab,kw OR (broncho alveolar carcinoma):ti,ab,kw OR (bronchioalveolar lung carcinoma):ti,ab,kw OR (bronchiolar cancer):ti,ab,kw (Word variations have been searched) 61

#21 (bronchiolar cell cancer):ti,ab,kw OR (bronchiolar cell carcinoma):ti,ab,kw OR (bronchoalveolar carcinoma):ti,ab,kw OR (bronchoalveolar cancer):ti,ab,kw OR (bronchial carcinomata):ti,ab,kw (Word variations have been searched) 425

#22 (broncho-pulmonary carcinoma):ti,ab,kw OR (bronchogenic carcinoma of the lung):ti,ab,kw OR (bronchogenic lung carcinoma):ti,ab,kw OR (bronchopulmonary carcinoma):ti,ab,kw OR (bronchus carcinomacarcinoma, bronchiolar):ti,ab,kw (Word variations have been searched) 250

#23 (bronchopulmonary cancer):ti,ab,kw OR (broncho-pulmonary neoplasm):ti,ab,kw OR (broncho-pulmonary tumor):ti,ab,kw OR (bronchopulmonary neoplasia):ti,ab,kw OR (bronchopulmonary neoplasm):ti,ab,kw (Word variations have been searched) 57

#24 (bronchopulmonary tumorCa lung):ti,ab,kw OR (bronchial small cell cancer):ti,ab,kw OR (bronchial small cell carcinoma):ti,ab,kw OR (bronchial non small cell cancer):ti,ab,kw OR (bronchial non small cell carcinoma):ti,ab,kw (Word variations have been searched) 256

#25 (Cancer, Lung):ti,ab,kw OR (Cancers, Lung):ti,ab,kw OR (Cancer, Pulmonary):ti,ab,kw OR (Cancers, Pulmonary):ti,ab,kw OR (Cancer of the Lung):ti,ab,kw (Word variations have been searched) 30820

#26 (Cancer of Lung):ti,ab,kw OR (cancer of the respiratory tract):ti,ab,kw OR (cancer in the thorax):ti,ab,kw OR (cancer of the thoracic cavity):ti,ab,kw OR (cancer of the thorax):ti,ab,kw (Word variations have been searched) 30263

#27 (cancer of the thorax and thoracic cavity):ti,ab,kw OR (cancerous lung cell line):ti,ab,kw OR (cancerous pulmonary cell line):ti,ab,kw OR (Carcinoma, Alveolar):ti,ab,kw OR (Carcinomas, Alveolar):ti,ab,kw (Word variations have been searched) 5073

#28 (Carcinoma, Alveolar Cell):ti,ab,kw OR (Carcinomas, Alveolar Cell):ti,ab,kw OR (Carcinoma, Bronchial):ti,ab,kw OR (Carcinomas, Bronchial):ti,ab,kw OR (Carcinoma, Bronchiolar):ti,ab,kw (Word variations have been searched) 417

#29 (Carcinomas, Bronchiolar):ti,ab,kw OR (Carcinoma, Bronchogenic):ti,ab,kw OR (Carcinomas, Bronchogenic):ti,ab,kw OR (Carcinoma, Bronchiolo-Alveolar):ti,ab,kw OR (Carcinoma, Bronchiolo Alveolar):ti,ab,kw (Word variations have been searched) 343

#30 (Carcinomas, Bronchiolo-Alveolar):ti,ab,kw OR (Carcinoma, Bronchioloalveolar):ti,ab,kw OR (Carcinomas, Bronchioloalveolar):ti,ab,kw OR (Carcinoma, Non-Small-Cell Lung):ti,ab,kw OR (Carcinoma, Non Small Cell Lung):ti,ab,kw (Word variations have been searched) 7199

#31 (Carcinomas, Non-Small-Cell Lung):ti,ab,kw OR (Carcinoma, Non-Small Cell Lung):ti,ab,kw OR (Carcinoma, Non-Small-Cell Lung):ti,ab,kw OR (Carcinoma, Non Small Cell Lung):ti,ab,kw OR (Carcinomas, Non-Small-Cell Lung):ti,ab,kw (Word variations have been searched) 7190

#32 (Carcinoma, Non-Small Cell Lung):ti,ab,kw OR (Carcinoma, Oat Cell):ti,ab,kw OR (Carcinomas, Oat Cell):ti,ab,kw OR (Carcinoma, Small Cell Lung):ti,ab,kw OR (Carcinoma, Small Cell):ti,ab,kw (Word variations have been searched) 9509

#33 (Carcinomas, Small Cell):ti,ab,kw OR (carcinoma of the thorax):ti,ab,kw OR (carcinoma of lung):ti,ab,kw OR (carcinoma of the lung):ti,ab,kw OR (carcinoma of the bronchus):ti,ab,kw (Word variations have been searched) 11748

#34 (carcinoma of bronchus):ti,ab,kw OR (carcinoma, lewis):ti,ab,kw OR (carcinoma, lewis lung):ti,ab,kw OR (carcinoma, lung):ti,ab,kw OR (carcinoma pulmonum):ti,ab,kw (Word variations have been searched) 10616

#35 (carcinomatosis of the lung):ti,ab,kw OR (carcinogenesis in the respiratory tract):ti,ab,kw OR (carcinogenesis of the lungbroncho-pulmonary cancer):ti,ab,kw OR (carcinomatous lung):ti,ab,kw OR (carcinomatous pulmonary):ti,ab,kw (Word variations have been searched) 82

#36 (invasive lung carcinoma):ti,ab,kw OR (invasive pulmonary carcinoma):ti,ab,kw OR (Lung Adenocarcinoma):ti,ab,kw OR (Lung Adenocarcinomas):ti,ab,kw OR (Lung Cancer):ti,ab,kw (Word variations have been searched) 29988

#37 (Lung Cancers):ti,ab,kw OR (Lung Carcinoma, Non-Small-Cell):ti,ab,kw OR (Lung Carcinomas, Non-Small-Cell):ti,ab,kw OR (Lung Neoplasm):ti,ab,kw OR (Lung Neoplasms):ti,ab,kw (Word variations have been searched) 31511

#38 (Lung Nodule, Solitary):ti,ab,kw OR (lung alveolar carcinoma):ti,ab,kw OR (lung alveolus cell carcinoma):ti,ab,kw OR (lung alveolar cell cancer):ti,ab,kw OR (lung alveolar cell carcinoma):ti,ab,kw (Word variations have been searched) 266

#39 (lung alveolus cell cancer):ti,ab,kw OR (lung cancer cell line):ti,ab,kw OR (lung cancer-derived cell line):ti,ab,kw OR (lung cancerous cell line):ti,ab,kw OR (lung cancers cell line):ti,ab,kw (Word variations have been searched) 4983

#40 (lung carcinoma):ti,ab,kw OR (lung carcinomata):ti,ab,kw OR (lung carcinomatosis):ti,ab,kw OR (lung cavitary carcinoma):ti,ab,kw OR (lung carcinogenesis):ti,ab,kw (Word variations have been searched) 10686

#41 (lung cancerogenesis):ti,ab,kw OR (microcellular lung carcinoma):ti,ab,kw OR (lung cancermalignancies of the lung):ti,ab,kw OR (lung cancer, non small cell):ti,ab,kw OR (lung malignancy):ti,ab,kw (Word variations have been searched) 18100

#42 (lung malignancies):ti,ab,kw OR (lung neoplasia):ti,ab,kw OR (lung non small cell cancer):ti,ab,kw OR (lung non small cell carcinoma):ti,ab,kw OR (lung oat cell carcinoma):ti,ab,kw (Word variations have been searched) 18660

#43 (lung small cell carcinoma):ti,ab,kw OR (lung small cell cancer):ti,ab,kw OR (lung tumor):ti,ab,kw OR (lung tumour):ti,ab,kw OR (lung tumorigenesis):ti,ab,kw (Word variations have been searched) 23336

#44 (lewis lung carcinoma):ti,ab,kw OR (lewis lung tumor):ti,ab,kw OR (lewis lung tumour):ti,ab,kw OR (Multiple Pulmonary Nodule):ti,ab,kw OR (Multiple Pulmonary Nodules):ti,ab,kw (Word variations have been searched) 172

#45 (malignancy of the lung):ti,ab,kw OR (malignant lung neoplasm):ti,ab,kw OR (malignant lung tumor):ti,ab,kw OR (malignant neoplasm of the lung):ti,ab,kw OR (malignant tumor of the lung):ti,ab,kw (Word variations have been searched) 3875

#46 (malignancies of the respiratory tract):ti,ab,kw OR (malignant lung cell line):ti,ab,kw OR (malignant pulmonary cell line):ti,ab,kw OR (Neoplasm, Bronchial):ti,ab,kw OR (Neoplasms, Bronchial):ti,ab,kw (Word variations have been searched) 1304

#47 (Neoplasm, Lung):ti,ab,kw OR (Neoplasms, Lung):ti,ab,kw OR (Neoplasm, Pulmonary):ti,ab,kw OR (Neoplasms, Pulmonary):ti,ab,kw OR (Neoplasm, Respiratory Tract):ti,ab,kw (Word variations have been searched) 15177

#48 (Neoplasm, Respiratory System):ti,ab,kw OR (Neoplasm, Respiratory):ti,ab,kw OR (Neoplasm, Tracheal):ti,ab,kw OR (Neoplasms, Tracheal):ti,ab,kw OR (Neoplasm, Thoracic):ti,ab,kw (Word variations have been searched) 3479

#49 (Neoplasms, Thoracic):ti,ab,kw OR (non small cell lung cancer):ti,ab,kw OR (Non-Small Cell Lung Cancer):ti,ab,kw OR (Non Small Cell Lung Carcinoma):ti,ab,kw OR (Non-Small-Cell Lung Carcinoma):ti,ab,kw (Word variations have been searched) 17057

#50 (Non-Small-Cell Lung Carcinomas):ti,ab,kw OR (Non-Small Cell Lung Carcinoma):ti,ab,kw OR (Non-Small-Cell Lung Carcinoma):ti,ab,kw OR (Non-Small Cell Lung Carcinoma):ti,ab,kw OR (Nonsmall Cell Lung Cancer):ti,ab,kw (Word variations have been searched) 13143

#51 (Nonsmall Cell Lung Cancer):ti,ab,kw OR (non oat cell lung cancer):ti,ab,kw OR (non small cell bronchial cancer):ti,ab,kw OR (non small cell cancer, lung):ti,ab,kw OR (non small cell pulmonary cancer):ti,ab,kw (Word variations have been searched) 15448

#52 (non small cell pulmonary carcinoma):ti,ab,kw OR (non squamous NSCLC):ti,ab,kw OR (nonsmall cell carcinoma of the lung):ti,ab,kw OR (nonsmall cell lung carcinoma):ti,ab,kw OR (Nodule, Solitary Lung):ti,ab,kw (Word variations have been searched) 6014

#53 (Nodule, Solitary Pulmonary):ti,ab,kw OR (Nodules, Solitary Pulmonary):ti,ab,kw OR (neoplasia of the lung):ti,ab,kw OR (neoplastic lung):ti,ab,kw OR (Oat Cell Carcinoma of Lung):ti,ab,kw (Word variations have been searched) 582

#54 (Oat Cell Lung Cancer):ti,ab,kw OR (Oat Cell Carcinoma):ti,ab,kw OR (Oat Cell Carcinomas):ti,ab,kw OR (oat cell cancer of the lung):ti,ab,kw OR (oat cell carcinoma of the lung):ti,ab,kw (Word variations have been searched) 73

#55 (oat cell lung carcinoma):ti,ab,kw OR (Pulmonary Cancer):ti,ab,kw OR (Pulmonary Cancers):ti,ab,kw OR (Pulmonary Neoplasm):ti,ab,kw OR (Pulmonary Neoplasms):ti,ab,kw (Word variations have been searched) 5029

#56 (Pulmonary Nodule, Solitary):ti,ab,kw OR (Pulmonary Nodules, Solitary):ti,ab,kw OR (Pulmonary Nodule, Multiple):ti,ab,kw OR (Pulmonary Nodules, Multiple):ti,ab,kw OR (pulmonary alveolar cell cancer):ti,ab,kw (Word variations have been searched) 279

#57 (pulmonary alveolus cell cancer):ti,ab,kw OR (pulmonary cancer cell line):ti,ab,kw OR (pulmonary cancerogenesis):ti,ab,kw OR (pulmonary carcinogenesis):ti,ab,kw OR (peribronchial carcinoma):ti,ab,kw (Word variations have been searched) 224

#58 (pulmonary carcinoma):ti,ab,kw OR (pulmonary carcinomatosis):ti,ab,kw OR (pulmonary malignancies):ti,ab,kw OR (pulmonary malignancy):ti,ab,kw OR (pulmonary neoplasia):ti,ab,kw (Word variations have been searched) 2204

#59 (pulmonary non small cell cancer):ti,ab,kw OR (pulmonary non small cell carcinoma):ti,ab,kw OR (pulmonary small cell cancer):ti,ab,kw OR (pulmonary small cell carcinomapulmonary tumor):ti,ab,kw OR (pulmonary tumorigenesis):ti,ab,kw (Word variations have been searched) 834

#60 (pulmonary tumour):ti,ab,kw OR (Respiratory Tract Neoplasms):ti,ab,kw OR (Respiratory Tract Neoplasm):ti,ab,kw OR (Respiratory System Neoplasms):ti,ab,kw OR (Respiratory System Neoplasm):ti,ab,kw (Word variations have been searched) 3112

#61 (Respiratory Neoplasms):ti,ab,kw OR (Respiratory Neoplasm):ti,ab,kw OR (respiratory tract cancer):ti,ab,kw OR (respiratory cancer):ti,ab,kw OR (respiratory carcinogenesis):ti,ab,kw (Word variations have been searched) 4536

#62 (respiratory malignancy):ti,ab,kw OR (respiratory malignancies):ti,ab,kw OR (respiratory tract carcinogenesis):ti,ab,kw OR (respiratory tract malignancies):ti,ab,kw OR (respiratory tract malignancy):ti,ab,kw (Word variations have been searched) 1223

#63 (respiratory tract carcinoma):ti,ab,kw OR (respiratory system carcinoma):ti,ab,kw OR (Small Cell Lung Cancer):ti,ab,kw OR (Small Cell Cancer Of The Lung):ti,ab,kw OR (Small Cell Carcinoma):ti,ab,kw (Word variations have been searched) 19659

#64 (Small Cell Carcinomas):ti,ab,kw OR (Small Cell Lung Carcinoma):ti,ab,kw OR (Solitary Pulmonary Nodule):ti,ab,kw OR (Solitary Pulmonary Nodules):ti,ab,kw OR (Solitary Lung Nodule):ti,ab,kw (Word variations have been searched) 9625

#65 (Solitary Lung Nodules):ti,ab,kw OR (small cell bronchial cancer):ti,ab,kw OR (small cell bronchial carcinoma):ti,ab,kw OR (small cell cancer, lung):ti,ab,kw OR (small cell carcinoma of the lung):ti,ab,kw (Word variations have been searched) 18377

#66 (small cell lung tumor):ti,ab,kw OR (small cell neuroendocrine carcinoma of the lung):ti,ab,kw OR (small cell pulmonary cancer):ti,ab,kw OR (small cell pulmonary carcinoma):ti,ab,kw OR (Tracheal Neoplasms):ti,ab,kw (Word variations have been searched) 7579

#67 (Tracheal Neoplasm):ti,ab,kw OR (Thoracic Neoplasm):ti,ab,kw OR (Thoracic Neoplasms):ti,ab,kw OR (tumor of the lung):ti,ab,kw OR (tumor, lung):ti,ab,kw (Word variations have been searched) 13255

#68 (tumorigenesis in the lung):ti,ab,kw OR (tumour, lung):ti,ab,kw OR (thoracic cancer):ti,ab,kw OR (thoracic carcinoma):ti,ab,kw OR (thoracic malignancies):ti,ab,kw (Word variations have been searched) 14714

#69 (thoracic malignancy):ti,ab,kw OR (thoracic malignant neoplasm):ti,ab,kw OR (thoracic malignant tumor):ti,ab,kw OR (thoracic sarcoma):ti,ab,kw OR (thorax cancer):ti,ab,kw (Word variations have been searched) 2536

#70 #13 OR #14 OR #15 OR #16 OR #17 OR #18 OR #19 OR #20 OR #21 OR #22 OR #23 OR #24 OR #25 OR #26 OR #27 OR #28 OR #28 OR #29 OR #30 OR #31 OR #32 OR #33 OR #34 OR #35 OR #36 OR #37 OR #38 OR #39 OR #40 OR #41 OR #42 OR #43 OR #44 OR #45 OR #46 OR #47 OR #48 OR #49 OR #50 OR #51 OR #52 OR #53 OR #54 OR #55 OR #56 OR #57 OR #58 OR #59 OR #60 OR #61 OR #62 OR #63 OR #64 OR #65 OR #66 OR #67 OR #68 OR #69 41565

#71 #12 OR #70 42196

#72 MeSH descriptor: [Deep Learning] explode all trees 264

#73 MeSH descriptor: [Algorithms] explode all trees 7045

#74 MeSH descriptor: [Artificial Intelligence] explode all trees 2832

#75 MeSH descriptor: [Machine Learning] explode all trees 880

#76 MeSH descriptor: [Supervised Machine Learning] explode all trees 104

#77 MeSH descriptor: [Unsupervised Machine Learning] explode all trees 5

#78 #72 OR #73 OR #74 OR #75 OR #76 OR #77 7045

#79 (Algorithms):ti,ab,kw OR (Algorithm):ti,ab,kw OR (Artificial Intelligence):ti,ab,kw OR (AI (Artificial Intelligence)):ti,ab,kw OR (AI):ti,ab,kw (Word variations have been searched) 24584

#80 (Acquisition, Knowledge (Computer)):ti,ab,kw OR (Active Machine Learning):ti,ab,kw OR (Automation):ti,ab,kw OR (Automations):ti,ab,kw OR (artificial neural network):ti,ab,kw (Word variations have been searched) 12180

#81 (artificial neural networks):ti,ab,kw OR (algorithmic neural network):ti,ab,kw OR (ANN (artificial neural network)):ti,ab,kw OR (ANN analysis):ti,ab,kw OR (ANN approach):ti,ab,kw (Word variations have been searched) 1864

#82 (ANN method):ti,ab,kw OR (ANN methodology):ti,ab,kw OR (ANN methods):ti,ab,kw OR (ANN model):ti,ab,kw OR (ANN models):ti,ab,kw (Word variations have been searched) 1417

#83 (ANN modeling):ti,ab,kw OR (ANN modelling):ti,ab,kw OR (ANN output):ti,ab,kw OR (ANN technique):ti,ab,kw OR (ANN techniques):ti,ab,kw (Word variations have been searched) 505

#84 (ANN training):ti,ab,kw OR (ANNs (artificial neural networks)):ti,ab,kw OR (artificial NN):ti,ab,kw OR (artificial NNs):ti,ab,kw OR (Computer Neural Network):ti,ab,kw (Word variations have been searched) 1153

#85 (Computer Neural Networks):ti,ab,kw OR (Computer Reasoning):ti,ab,kw OR (Computer Vision Systems):ti,ab,kw OR (Computer Vision System):ti,ab,kw OR (Computational Neural Networks):ti,ab,kw (Word variations have been searched) 3892

#86 (Computational Neural Network):ti,ab,kw OR (computerized neural network):ti,ab,kw OR (Computing Methodologies):ti,ab,kw OR (Computing Methodology):ti,ab,kw OR (Computing, High Performance):ti,ab,kw (Word variations have been searched) 9377

#87 (Connectionist Model):ti,ab,kw OR (Computational Intelligence):ti,ab,kw OR (Connectionist Models):ti,ab,kw OR (connectionist network):ti,ab,kw OR (connectionist neural network):ti,ab,kw (Word variations have been searched) 974

#88 (connectionist system):ti,ab,kw OR (convolutional neural network):ti,ab,kw OR (CNN (convolutional neural network)):ti,ab,kw OR (CNNs (convolutional neural networks)):ti,ab,kw OR (ConvNet):ti,ab,kw (Word variations have been searched) 543

#89 (convoluted neural network):ti,ab,kw OR (convolution neural network):ti,ab,kw OR (convolutional ANNs):ti,ab,kw OR (convolutional artificial neural network):ti,ab,kw OR (convolutional deep neural network):ti,ab,kw (Word variations have been searched) 541

#90 (convolutional NN):ti,ab,kw OR (convolutionary neural network):ti,ab,kw OR (convolution algorithm):ti,ab,kw OR (convolutional algorithm):ti,ab,kw OR (convolutionary algorithm):ti,ab,kw (Word variations have been searched) 254

#91 (convolution superposition algorithm):ti,ab,kw OR (convolution superposition algorithm):ti,ab,kw OR (convolutionsuperposition algorithm):ti,ab,kw OR (U Net):ti,ab,kw OR (V Net):ti,ab,kw (Word variations have been searched) 949

#92 (CNN):ti,ab,kw OR (Deep Learning):ti,ab,kw OR (DNN (deep neural network)):ti,ab,kw OR (DNNs (deep neural networks)):ti,ab,kw OR (deep convolutional neural network):ti,ab,kw (Word variations have been searched) 1577

#93 (deep neural network):ti,ab,kw OR (deep artificial neural network):ti,ab,kw OR (deep NNs):ti,ab,kw OR (feature-based learning):ti,ab,kw OR (feature learning (machine learning)):ti,ab,kw (Word variations have been searched) 1326

#94 (feature learning (machine learning)):ti,ab,kw OR (Hierarchical Learning):ti,ab,kw OR (High Performance Computing):ti,ab,kw OR (Intelligence, Artificial):ti,ab,kw OR (Intelligence, Computational):ti,ab,kw (Word variations have been searched) 9432

#95 (Intelligence, Machine):ti,ab,kw OR (Inductive Machine Learning):ti,ab,kw OR (Knowledge Acquisition (Computer)):ti,ab,kw OR (Knowledge Representation (Computer)):ti,ab,kw OR (Knowledge Representations (Computer)):ti,ab,kw (Word variations have been searched) 621

#96 (Learning, Deep):ti,ab,kw OR (Learning, Hierarchical):ti,ab,kw OR (Learning, Machine):ti,ab,kw OR (learning machines):ti,ab,kw OR (Learning, Active Machine):ti,ab,kw (Word variations have been searched) 3974

#97 (Learning, Supervised Machine):ti,ab,kw OR (Learning, Unsupervised Machine):ti,ab,kw OR (Learning, Semi-supervised):ti,ab,kw OR (Learning from Labeled Data):ti,ab,kw OR (Learning, Transfer):ti,ab,kw (Word variations have been searched) 2612

#98 (Learning, Inductive Machine):ti,ab,kw OR (Methodologies, Computing):ti,ab,kw OR (Methodology, Computing):ti,ab,kw OR (Machine Intelligence):ti,ab,kw OR (Machine Learning):ti,ab,kw (Word variations have been searched) 4901

#99 (Machine Learning, Supervised):ti,ab,kw OR (Machine Learning, Unsupervised):ti,ab,kw OR (Machine Learning, Inductive):ti,ab,kw OR (Machine, Support Vector):ti,ab,kw OR (Machines, Support Vector):ti,ab,kw (Word variations have been searched) 709

#100 (Models, Neural Network):ti,ab,kw OR (Model, Neural Network):ti,ab,kw OR (Model, Connectionist):ti,ab,kw OR (Models, Connectionist):ti,ab,kw OR (Machine Learning, Active):ti,ab,kw (Word variations have been searched) 1562

#101 (Machine Learning with a Teacher):ti,ab,kw OR (mathematical neural network):ti,ab,kw OR (manifold learning):ti,ab,kw OR (Network, Support Vector):ti,ab,kw OR (Networks, Support Vector):ti,ab,kw (Word variations have been searched) 182

#102 (Network, Computer Neural):ti,ab,kw OR (Networks, Computer Neural):ti,ab,kw OR (Network, Neural (Computer)):ti,ab,kw OR (Network Model, Neural):ti,ab,kw OR (Network Models, Neural):ti,ab,kw (Word variations have been searched) 1553

#103 (Networks, Neural (Computer)):ti,ab,kw OR (Network, Computational Neural):ti,ab,kw OR (Networks, Computational Neural):ti,ab,kw OR (Neural Network, Computer):ti,ab,kw OR (Neural Network Model):ti,ab,kw (Word variations have been searched) 1553

#104 (Neural Network Models):ti,ab,kw OR (Neural Network, Computational):ti,ab,kw OR (Neural Networks, Computational):ti,ab,kw OR (Neural Networks, Computer):ti,ab,kw OR (Neural Network (Computer)):ti,ab,kw (Word variations have been searched) 1553

#105 (Neural Networks (Computer)):ti,ab,kw OR (Neural Network (Computer)Neural Networks, Computer):ti,ab,kw OR (neural network (artificial)):ti,ab,kw OR (neural network algorithm):ti,ab,kw OR (Perceptrons):ti,ab,kw (Word variations have been searched) 1338

#106 (Perceptron):ti,ab,kw OR (Performance Computing, High):ti,ab,kw OR (Representation, Knowledge (Computer)):ti,ab,kw OR (Reasoning, Computer):ti,ab,kw OR (representation learning):ti,ab,kw (Word variations have been searched) 9520

#107 (representation-based learning):ti,ab,kw OR (System, Computer Vision):ti,ab,kw OR (Systems, Computer Vision):ti,ab,kw OR (Semi-supervised Learning):ti,ab,kw OR (Semi supervised Learning):ti,ab,kw (Word variations have been searched) 433

#108 (Supervised Machine Learning):ti,ab,kw OR (Support Vector Machine):ti,ab,kw OR (Support Vector Machines):ti,ab,kw OR (Support Vector Network):ti,ab,kw OR (Support Vector Networks):ti,ab,kw (Word variations have been searched) 631

#109 (superposition convolution algorithm):ti,ab,kw OR (superposition convolution algorithm):ti,ab,kw OR (superpositionconvolution algorithm):ti,ab,kw OR (Transfer Learning):ti,ab,kw OR (Unsupervised Machine Learning):ti,ab,kw (Word variations have been searched) 2109

#110 (Vision System, Computer):ti,ab,kw OR (Vision Systems, Computer):ti,ab,kw OR (Vector Machine, Support):ti,ab,kw OR (Vector Machines, Support):ti,ab,kw OR (Vector Network, Support):ti,ab,kw (Word variations have been searched) 845

#111 (Vector Networks, Support):ti,ab,kw (Word variations have been searched) 123

#112 #79 OR #80 OR #81 OR #82 OR #83 OR #84 OR #85 OR #86 OR #87 OR #88 OR #89 OR #90 OR #91 OR #92 OR #93 OR #94 OR #95 OR #96 OR #97 OR #98 OR #99 OR #100 OR #101 OR #102 OR #103 OR #104 OR #105 OR #106 OR #107 OR #108 OR #109 OR #110 OR #111 51741

#113 #78 OR #112 52930

#114 (segment):ti,ab,kw OR (segmentation):ti,ab,kw (Word variations have been searched) 24881

#115 #71 AND #113 AND #114 100

**Risk of Bias Assessment:**

The detailed evaluation criteria:

**DOMAIN 1: PATIENT SELECTION**

**A. Risk of Bias**

Were the data sources clear, and are there clear criteria for inclusion and exclusion(ex: nodule size)?

**B. Concerns regarding applicability**

Were the parameters of lung nodule scanning fully known?

**DOMAIN 2: INDEX TEST(S)**

1. **Risk of Bias**

Was the training set, test set, or verification set specified?

**B. Concerns regarding applicability**

Was there an independent set of external validations?

**DOMAIN 3: REFERENCE STANDARD**

**A. Risk of Bias**

Was there any specific information that clearly states the reference standards?

**B. Concerns regarding applicability**

Was there an intersection of two (or more) expert sketches as ground truth?

**DOMAIN 4: FLOW AND TIMING**

**A. Risk of Bias**

Were deep learning algorithms explicitly described?

Supplementary Table 1 Methodological overview of the included studies

| **First author** | **Methodological overview of the included studies** |
| --- | --- |
| Huang, X  [33] | This article introduced a fast and fully-automated end-to-end system that can efficiently segment precise lung nodule contours from raw thoracic CT scans. The proposed system has four major modules: candidate nodule detection with Faster R-CNN, candidate merging, FP reduction with CNN, and nodule segmentation with customized FCN. The entire system has no human interaction or database specific design. |
| Cai, L  [34] | Aiming at assisting radiologists to diagnose pulmonary nodules more accurately, the methods of detection and segmentation for pulmonary nodule 3D visualization diagnosis were proposed based on Mask R-CNN and ray-casting volume rendering algorithm. The Mask R-CNN used resnet50 as the backbone and applied FPN to fully explore multiscale feature maps. And then, RPN was used to propose candidate bounding boxes. Furthermore, the mask matrices and the raw medical image sequences were multiplied to obtain sequences of predicted pulmonary nodules. Finally, ray-casting volume rendering algorithm was applied to generate the 3D models of pulmonary nodules. |
| Banu, S. F  [37] | This article proposes a fully automated deep learning framework that consists of lung nodule detection and segmentation models. The proposed system comprises two cascaded stages: (1) nodule detection based on fine-tuned Faster R-CNN to localize the nodules in CT images, and (2) nodule segmentation based on the U-Net architecture with two effective blocks, namely position attention-aware weight excitation and channel attention-aware weight excitation, to enhance the ability to discriminate between nodule and non-nodule feature representations. |
| Dutande, P  [35] | This paper presents a novel approach for lung nodule classification, detection, and segmentation using a 2D-3D cascaded CNN architecture. The paper focuses on addressing the challenges in accurately segmenting lung nodules, such as the presence of similar structures and varying shapes and sizes. The proposed methodology includes the use of a modified version of the U-Net architecture called SquExU-Net for nodule segmentation. This architecture incorporates squeeze and excitation blocks to extract fine-grained information for accurate segmentation. Additionally, a 3D-NodNet classification model is introduced to classify nodule candidates obtained from the segmentation framework. |
| Zhang, X  [36] | In this study, an automatic detection and segmentation method for lung nodules in different locations has been developed. First, they apply Otsu thresholding to segment lung parenchyma. Next, a morphological opening operation is carried out to remove blood vessels. Then α-hull operation is proposed to correct lung contours and optimal α values can be acquired adaptively. Finally, DenseNet convolutional network is applied to classify true lung nodules from all nodule candidates. |
| Hesamian, M. H.[38] | This paper presents a method for the detection and segmentation of lung nodules in CT images using synthetic CT images. The authors propose a technique to capture the inter-slice information by creating synthetic pseudo-color images that represent the changes of nodules over continuous slices. They then use a deep learning-based image segmentation technique to segment the nodules from these synthetic images. The main contributions of the paper are the development of a new method to embed inter-slice information into synthetic color images, the creation of an end-to-end segmentation network for detecting and segmenting nodules directly from lung CT images, and the successful addressing of the class imbalance issue during network training using weighted loss. |
| Primakov, S. P.  [39] | This study utilized the 2D U-net CNN deep learning algorithm for the automatic detection and segmentation of non-small cell lung cancer. The original CNN architecture was enhanced to improve segmentation performance. The authors created a fully automated pipeline capable of accommodating various CT acquisition and reconstruction parameters, making it suitable for CT scans with different settings. |
| Zhou, Z  [40] | The article propose a 2.5D-based cascaded multi-stage framework for automatic detection and segmentation of pulmonary nodules. The first three stages of the framework are used to discover lesions, and the latter stage is used to segment them. The first locating stage introduces the classical 2D-based Yolov5 model to locate the nodules roughly on axial slices. The second aggregation stage proposes a candidate nodule selection CNS algorithm to locate further and reduce redundant candidate nodules. The third classification stage uses a multi-size 3D-based fusion model to accommodate nodules of varying sizes and shapes for false-positive reducing. The last segmentation stage introducesmulti-scale and attention modules into 3Dbased UNet autoencoder to segment the nodular regions finely. |
| Dlamini, S  [41] | The article aim to develop a fully automatic system that will detect, segment and accurately reconstruct non-small cell lung cancer tumors into space using YOLOv4 and region-based active contour model. The system consists of two main sections which are detection and volumetric rendering. The detection section is composed of image enhancement, augmentation, labeling and localization while the volumetric rendering is mainly image filtering, tumor extraction, region-based active contour and 3D reconstruction. In this method the images are enhanced to eliminate noise before augmentation which is intended to multiply and diversify the image data. Labeling was then carried out in order to create a solid learning foundation for the localization model. Images with localized tumors were passed through smoothing filters and then clustered to extract tumor masks. Lastly contour infor­ mation was obtained to render the volumetric tumor. |

***2D***, 2-dimensional; ***2.5D***, 2.5-dimensional; ***3D***, 3-dimensional; ***CNN***, convolutional neural network; ***CNS***, candidate nodule selection; ***FCN***, fully convolutional neural network; ***FP***, false positive; ***FPN***, Feature Pyramid Network; ***Mask R-CNN***, Mask Region-Convolutional Neural Network; ***RPN***, Region Proposal Network; ***R-CNN***, regional-CNN;
